# Persistent Impact of *In utero* Irradiation on Mouse Brain Structure and Function Characterized by MR Imaging and Behavioral Analysis

**DOI:** 10.3389/fnbeh.2016.00083

**Published:** 2016-05-04

**Authors:** Tine Verreet, Janaki Raman Rangarajan, Roel Quintens, Mieke Verslegers, Adrian C. Lo, Kristof Govaerts, Mieke Neefs, Liselotte Leysen, Sarah Baatout, Frederik Maes, Uwe Himmelreich, Rudi D'Hooge, Lieve Moons, Mohammed A. Benotmane

**Affiliations:** ^1^Laboratory of Molecular and Cellular Biology, Institute for Environment, Health and Safety, Belgian Nuclear Research Centre (SCK•CEN)Mol, Belgium; ^2^Laboratory of Neural Circuit Development and Regeneration, Animal Physiology and Neurobiology Section, Department of Biology, Faculty of Science, Katholieke Universiteit LeuvenLeuven, Belgium; ^3^Faculty of Medicine, Molecular Small Animal Imaging Center, Katholieke Universiteit LeuvenLeuven, Belgium; ^4^Department of Electrical Engineering (ESAT/PSI), Katholieke Universiteit Leuven and Medical Image Research Center, University Hospital LeuvenLeuven, Belgium; ^5^Laboratory of Biological Psychology, Faculty of Psychology and Educational Sciences, Katholieke Universiteit LeuvenLeuven, Belgium; ^6^Biomedical MRI Unit, Department of Imaging and Pathology, Faculty of Medicine, Katholieke Universiteit LeuvenLeuven, Belgium

**Keywords:** brain development, cognition, microcephaly, MRI, radiation exposure, sociability

## Abstract

Prenatal irradiation is known to perturb brain development. Epidemiological studies revealed that radiation exposure during weeks 8–15 of pregnancy was associated with an increased occurrence of mental disability and microcephaly. Such neurological deficits were reproduced in animal models, in which rodent behavioral testing is an often used tool to evaluate radiation-induced defective brain functionality. However, up to now, animal studies suggested a threshold dose of around 0.30 Gray (Gy) below which no behavioral alterations can be observed, while human studies hinted at late defects after exposure to doses as low as 0.10 Gy. Here, we acutely irradiated pregnant mice at embryonic day 11 with doses ranging from 0.10 to 1.00 Gy. A thorough investigation of the dose-response relationship of altered brain function and architecture following *in utero* irradiation was achieved using a behavioral test battery and volumetric 3D T_2_-weighted magnetic resonance imaging (MRI). We found dose-dependent changes in cage activity, social behavior, anxiety-related exploration, and spatio-cognitive performance. Although behavioral alterations in low-dose exposed animals were mild, we did unveil that both emotionality and higher cognitive abilities were affected in mice exposed to ≥0.10 Gy. Microcephaly was apparent from 0.33 Gy onwards and accompanied by deviations in regional brain volumes as compared to controls. Of note, total brain volume and the relative volume of the ventricles, frontal and posterior cerebral cortex, cerebellum, and striatum were most strongly correlated to altered behavioral parameters. Taken together, we present conclusive evidence for persistent low-dose effects after prenatal irradiation in mice and provide a better understanding of the correlation between their brain size and performance in behavioral tests.

## Introduction

Exposure to ionizing radiation is known to induce a plethora of adverse health consequences, with the severity of the effects greatly depending on the dose (Williams and Fletcher, 2010). Brain defects, including mental disability and microcephaly, have been reported in humans and laboratory animals *in utero* exposed to radiation (Schull et al., 1990; Schull and Otake, 1999). Apart from epidemiological evidence in atomic bomb survivors, the altered functionality of the brain following radiation exposure is typically demonstrated using rodent behavioral testing. Diverse behavioral paradigms have been employed and revealed alterations in cortical and hippocampal-associated functions, like changed exploration and anxiety (Tomášová et al., 2012; Kokošová et al., 2015), as well as impaired cognition (Sienkiewicz et al., 1999). For instance, X-ray exposure of the rat at embryonic day (E) 17 with 2.25 Gray (Gy), used to induce cortical dysplasia, affected the progeny's non-locomotor activity, motor coordination, and learning and memory (Zhou et al., 2011). Whether low doses of radiation (≤ 0.10 Gy) also disturb brain function is less clear. While evidence from epidemiological data hinted to a threshold dose of 0.10 Gy below which no long-term brain functional deficits could be observed (Otake et al., 1996), animal studies assessing the dose-response relationship of prenatal irradiation rather indicated that no behavioral effects could be seen below 0.30 Gy (Sienkiewicz et al., 1994; Hossain and Uma Devi, 2001). Indeed, aberrant open field and radial arm maze performance has been found in mice prenatally exposed to 0.30 and 0.35 Gy, but not in those exposed to 0.25 Gy (Baskar and Devi, 2000; Hossain and Uma Devi, 2000). In light of this discrepancy, the consequences of *in utero* radiation exposure to low doses < 0.30 Gy are a matter of debate and require further attention (Mothersill and Seymour, 2013; Doss et al., 2014).

In a previous study, we identified E11 as a critical time point for long-term radiation health effects (Verreet et al., 2015), including behavioral alterations and brain volumetric changes as assessed by non-invasive 3D T_2_-weighted magnetic resonance imaging (MRI). In the present report, we evaluated the dose-response relationship of mouse behavioral defects after radiation exposure at E11 with 0.10, 0.33, 0.66, or 1.00 Gy, aiming to better define the threshold, if one exists, of the functional and structural sequelae for the brain resulting from prenatal irradiation. Hereto, an extended behavioral test battery was used, including clinically relevant elements such as emotional response, exploration, and cognition. Subsequently, animals were subjected to MRI in order to assess possible volumetric/structural changes in the irradiated brains that could be related to the behavioral alterations. *In vivo* MRI has proven a valid tool in neuroscience research and has, for example, been used to analyze the adverse outcome of radiotherapy treatment on the brain (Atwood et al., 2007a,b; Chan et al., 2009) and regional differences in neuroanatomy and brain growth after 7-Gy whole-brain irradiation in 2.5-week-old mice (Gazdzinski et al., 2012). Registration-based methods have been widely used to assess brain volume changes in animal models of neurodegenerative disorders (Grand'maison et al., 2013; Sawiak et al., 2013). They have, however, never been utilized before to determine volumetric differences in the prenatally irradiated mouse brain. A preliminary report in non-human primates suggested that fractionated irradiation during prenatal neurogenesis caused a non-homogeneous reduction in gray matter volumes, particularly in the putamen, and cerebral cortex (Selemon et al., 2013). Volume decreases were not always significant, probably as a result of a low number of animals and a large within-group variability, but the brain volume changes were accompanied by functional deficits under the form of adult-onset defects in a working memory task (Aldridge et al., 2012; Selemon et al., 2013).

In this study, we investigated behavior in young adult mice that had been irradiated *in utero*. A dose of 0.10 Gy delivered at E11 caused altered sociability and compromised spatio-cognitive performance, whereas 1.00-Gy exposed animals exhibited decreased 23-h cage activity, defective anxiety-related exploration, sociability and social memory, as well as clear defects in spatial learning and memory. Moreover, mice irradiated with a dose of 0.33 Gy or higher displayed microcephaly. MRI further demonstrated changes in the absolute and relative volumes of certain brain regions. We performed correlation analyses between brain (region) volumes and radiation-induced behavioral alterations. This disclosed strong correlative relationships between the overall reduction in brain volume, decreased normalized posterior and frontal cortex, striatum and cerebellum volume, and increased relative ventricle size with various behavioral outcomes. For instance, radiation-induced altered Morris water maze (MWM) escape latency during training day 1 correlated significantly with changes in cortical and cerebellar volume. Hence, with this work, we contribute to a better understanding of the relationship between brain structure and function in prenatally irradiated mice and we also present evidence for decreased brain functionality due to *in utero* exposure to a radiation dose as low as 0.10 Gy.

## Materials and methods

### Animals and irradiation procedure

C57Bl/6J mice were purchased from Charles River Breeding Laboratories (Leiden, The Netherlands) and kept in standard animal cages under conventional laboratory conditions (12-h light/dark cycle, dark cycle starts at 20.00 h and 22°C), with *ad libitum* access to water and food. All experiments were performed in accordance with the European Communities Council Directive of 24 November 1986 (86/609/EEC) and approved by the local ethical committees at SCK•CEN (Request no. 11-005) and KU Leuven. Mating of mice occurred during a 2-h period in the morning. This short mating period was used in order to obtain synchronous timing of embryonic development. At E11, pregnant mice were whole-body-irradiated in a Plexiglas box with different doses (0.10, 0.33, 0.66, or 1.00 Gy). Irradiation was performed using a Pantak HF420 RX machine (Branford, CT, US) operating at 250 kV, 15 mA (1-mm Cu-filtered X-rays, dose rate of 0.375 Gy min^−1^). Doses were determined using a Farmer ionization chamber. Control mice were sham-irradiated. After irradiation, pregnant mice returned to their home cages and were allowed to give birth. Three weeks after birth, pups were weaned and litters were group-housed per gender for subsequent experiments.

### Behavioral tests

Neuromotor, exploration, and learning assessment started when animals were 4–7 weeks old and ended at 12–15 weeks of age. An overview of the order of behavioral tests and the age range of the animals utilized for every task is provided in Table 1. Female animals were used and testing occurred using two cohorts of mice, each containing control animals (*N* = 15). In order to correct for minor inter-experimental variation, behavioral parameters are expressed as a percentage in regard to their respective controls, except for MWM strategies.

**Table 1:** **Overview of test order and age at time of testing**.

**Protocol**	**Age range (weeks)**
Cage activity	4–7
Accelerating rotarod	5–8
Gait analysis	5–8
Open field	6–9
Social exploration	7–10
Elevated plus maze	8–11
Sociability and preference for social novelty (SPSN)	9–12
Morris water maze (MWM)	10–15

#### Cage activity

Ambulatory cage activity was recorded with a laboratory-built activity logger connected to three infrared beams. Mice were placed individually in a transparent cage (20 × 26 cm) during 23 h and beam crossings representing locomotor cage activity were registered during 30-min time intervals.

#### Accelerating rotarod

Motor coordination and equilibrium were tested on an accelerating rotarod apparatus (MED Associates Inc., St. Albans, VT, US). After two adaptation trials of 2 min each at a constant speed of 4 rpm, a mouse was placed on the rotating rod for four test trials with an inter-trial interval of 10 min. The time (latency) a mouse could remain on the rod was measured up to a maximum of 5 min, during which rotation speed gradually increased from 4 to 40 rpm.

#### Gait analysis

Walking abilities and gait pattern were analyzed using the DigiGait Imaging System (Mouse Specifics, Boston, MA, US). Mice were placed on a motor-driven controlled treadmill with a constant speed of 22 cm s^−1^ and filmed with a digital camera that was placed underneath a transparent belt. The digital images of the gait pattern were used to extract gait parameters for each mouse. Variables considered were: stride length, paw angle, absolute paw angle, stance/swing ratio, hind limb shared stance time, stance, stride, and swing.

#### Open field

Open field exploration was examined in an illuminated 50 × 50 cm square Plexiglas arena. After a minimum of 30-min dark adaptation, every animal was placed in the same corner of the arena and allowed to adapt to the arena during 1 min. Thereafter, movement of the mouse in the arena was recorded during 10 min with ANY-maze TM video tracking equipment and software (Stoelting Co., IL, US). Total path length, mean speed and line crossings were included as measures of ambulatory activity. Center (defined as a circle with 30-cm diameter in the middle of the arena) entries, percentage of path length in the center, latency of first center approach, and time spent in the center were used to evaluate exploration and anxiety-related behaviors.

#### Social exploration

Social interaction is another method to measure anxiety-related behavior (File and Seth, 2003). To assess social exploration, the same arena was used as in the open field task. A round wire cage with two female stranger mice (STR) was placed in the center of the arena, enabling visual, olfactory, and limited physical contact with the test animal. Mice started from a specific corner of the arena and recording of the explorative pattern began after 1 min of adaptation. During the following 10 min, the number of center approaches, latency of first center approach, percentage of path length in the center, and time spent in the center were recorded. Total path length, mean speed, and line crossings were included as measures of ambulatory activity.

#### Elevated plus maze

The elevated plus maze was used to evaluate anxiety-related exploration. Mice could first adapt for 1 min in the plus-shaped maze, located 30 cm above the surface. The maze consisted of two open arms (21 × 5 cm) without walls and two closed arms of the same size with high-side walls. After adaptation, mice could explore the maze freely for 10 min during which exploratory activity was recorded by five infrared beams (four for arm entries and one for percentage of time spent in the open arms) connected to a computerized activity logger. Total number of arm entries (beam crossings in open and closed arms), open arm entries, closed arm entries, percentage of open arm entries, percentage of time spent in the open arms, and ratio open/closed arm entries were used as parameters.

#### Sociability and preference for social novelty (SPSN)

The Sociability and Preference for Social Novelty (SPSN) procedure was performed as described previously (Naert et al., 2011). The apparatus consisted of an indirectly-illuminated transparent Plexiglas box with one central chamber and two adjacent chambers. The adjacent chambers were accessible through a small opening that could be manually closed. In each of the adjacent chambers, a round wire cage was placed. ANY-maze was used to record the animal's behavior. The procedure consisted of three trials and between trials the mouse was removed from the set-up and placed separately in a waiting cage. During the first trial (acclimation trial), mice could freely explore the central chamber for 5 min, doors to the adjacent chambers remained closed. In the second trial (sociability trial), an unknown female mouse (STR1) was placed in one of the wire cages while the other wire cage remained empty. The doors to all chambers were opened shortly after the start of the trial and mice could walk around freely for 10 min while their behavior was recorded. For the last trial (preference for social novelty trial), a second unknown female mouse (STR2) was placed in the empty wire cage; STR1 remained in its place. Similar to the second trial, the test mouse was placed in the central chamber and after the start of the trial the doors to all chambers were opened (10 min). Approach behavior toward the STR mice by the test animal was defined as the time spent and the amount of entries in a circle of 1.4 cm around the wire cages. The latency of the first approach per STR and the total path length in each chamber were also registered. The position of STR1 and STR2 was counterbalanced between test animals.

#### Morris water maze (MWM)

Spatial learning and memory were examined in the MWM. A round acrylic glass platform (15-cm diameter) with a fixed position was hidden 1 cm below the surface of opacified water, kept at 25–26°C. The circular pool had a diameter of 150 cm (and height of 30 cm) and the room housing the pool had a constant display of distal extra-maze cues. Swimming patterns of the animals were recorded with EthoVision video tracking equipment and software (Noldus, Wageningen, The Netherlands). During the acquisition phase, mice had to swim four times daily with a trial interval of 10–15 min, starting randomly from one of four starting positions. Mice that failed to find the platform within 2 min were gently guided to the platform and remained there for 10–15 s before they were returned to their home cage. Animals were trained during two blocks of 5 days (2 days of rest followed after each training block). Escape latency to find the hidden platform, path length, and swimming velocity were recorded.

Differential search strategies, as defined previously by Brody and Holtzman (2006), were assigned to each trial. Strategies can be classified into three classes, each with subcategories: (1) spatial: spatial direct, spatial indirect, focal correct; (2) non-spatial systematic: scanning, random, focal incorrect; and (3) repetitive looping: chaining, peripheral looping, circling (see also Figure S1). The strategy predominantly used by the animal was attributed for each trial and the amount of trials per day in each strategy was counted and expressed as a percentage (Callaerts-Vegh et al., 2012; Lo et al., 2014).

Probe trials were performed on day 6 and 11. During these probe trials, the platform was removed and the search pattern of the animals was recorded during 100 s. Time spent in each quadrant, as well as path length, and swimming velocity, were recorded.

### MR imaging

#### Image acquisition

In order to assess neuropathological consequences of prenatal radiation exposure *in vivo*, anatomical MRI was performed for all animals at the average age of 22 weeks. For this purpose, we used the same mice as for the behavioral tests. A 9.4-Tesla Bruker Biospec MR scanner (20-cm horizontal bore and 600 mT m^−1^-actively shielded gradients, Bruker Biospin, Ettlingen, Germany) was used with a 7.2-cm linearly-polarized resonator for transmission and a dedicated mouse brain surface receive coil (both Bruker Biospin) to obtain 3D high resolution images of the entire mouse brain (acquisition time: 20 min). Localizer images (2D, multi-slice) were acquired first and used for proper positioning and orientation of subsequently-acquired 3D MR images. We used a conventional 3D RARE (rapid acquisition with refocused echoes) sequence with the following parameters: repetition time = 1.3 s, echo time = 14.2 ms, and an isotropic resolution of 80 μm. Animals were anesthetized by inhalation of 3–4% isoflurane in 100% oxygen (Abbott Laboratories, Queenborough, UK) for induction and 1–2% isoflurane for maintenance. Mouse body temperature and breathing rate were monitored continuously using an MR-compatible physiological monitoring system (SAII, Stony Brook, NY, US) and maintained at 35–37°C and 140 min^−1^, respectively.

#### Image post-processing

For morphological characterization of mouse brain MRI, a dedicated semi-automated image analysis pipeline was used (Kovacevic et al., 2005; Cleary et al., 2011; Ma et al., 2014), consisting of three main stages: (1) pre-processing, (2) study-specific template construction, and (3) atlas-based volumetry.

In the first stage, the study images were pre-processed to correct for intra-scan inhomogeneity (Likar et al., 2001). The *ex vivo* LONI atlas (MacKenzie-Graham et al., 2004) was subsequently affinely aligned with each of the corrected study images using automated image registration (Maes et al., 1997). Based on the LONI atlas, a brain mask, dilated with three voxels, was defined for each of the study images. Image intensities within the brain masks were linearly normalized across subjects. The intensity-normalized MR images and corresponding brain masks were used in all subsequent steps.

In the second stage, the study images of both control and irradiated animals were spatially normalized by free-form non-rigid registration using B-splines (Rueckert et al., 1999), as implemented in NiftyReg (Modat et al., 2010), in order to iteratively construct a study-specific mean shape template. In the first iteration, all images were rigidly aligned (three translation and three rotation parameters) to the publicly available *in vivo* NUS atlas (Bai et al., 2012) that is pre-aligned to the LONI atlas. In the second iteration, all images were affinely registered (three translation, three rotation, three scale, and three shear parameters) to their average from the first iteration. In the third iteration, all images were non-rigidly registered to their average from the second iteration [final B-spline control point spacing of 400 μm (five voxels)]. This step was repeated 10 times, whereby at each time the average of the previous iteration was geometrically centered to the study population by subtracting the mean displacement over all subjects. Anatomical labels were assigned to the final template through its non-rigid registration to the NUS atlas (39 anatomical labels, for details see Bai et al., 2012).

**Figure 1:**
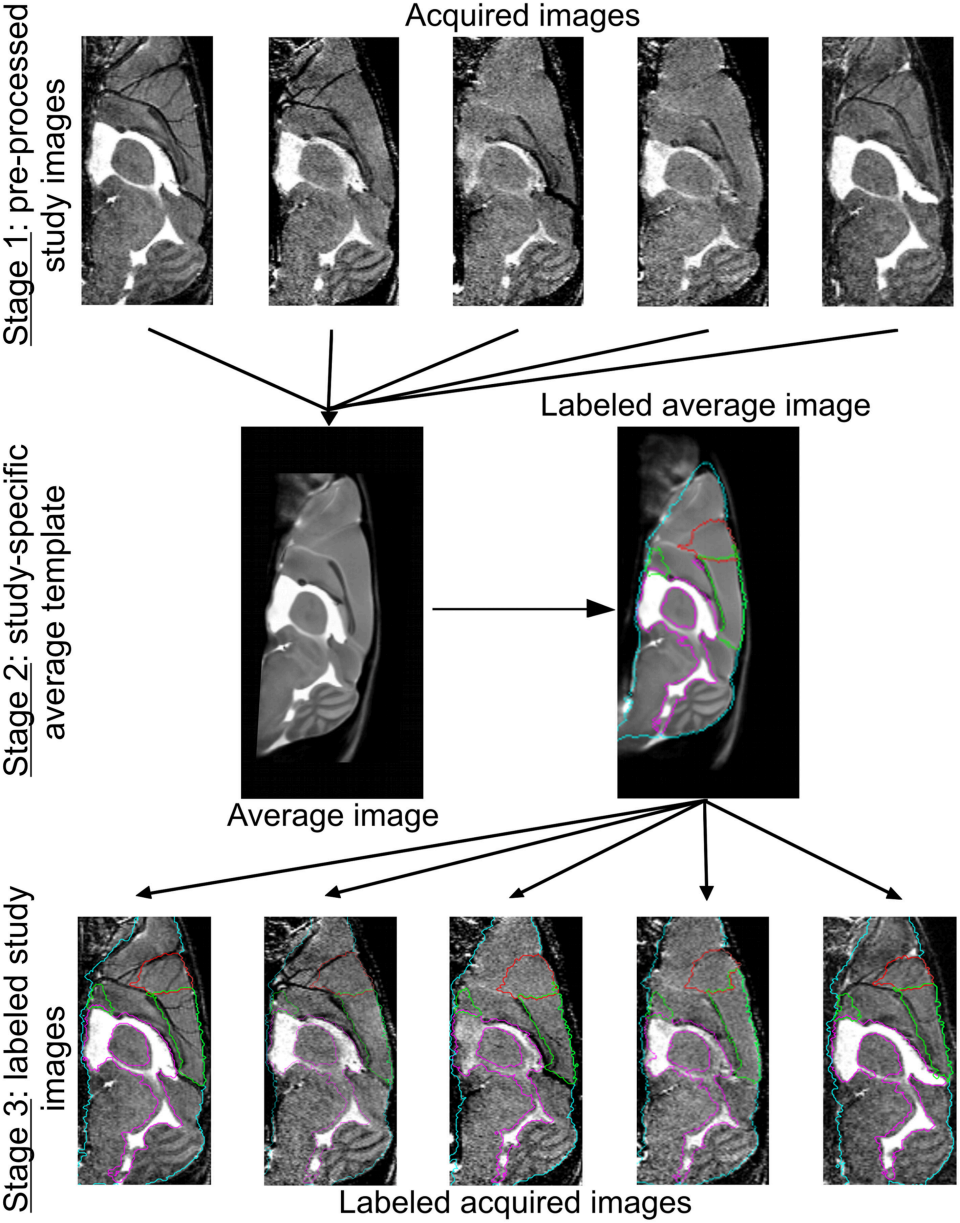
**Image registration and labeling**. On top, five representative brain MR images are shown. Of all the images from all conditions, we prepared an average study template, which was then registered to the NUS atlas in order to segment the brain into specific brain regions. These brain structures or labels were propagated to the individual images of every animal. The whole brain contour is shown in cyan, the ventricles are delineated in purple, and the frontal and cerebral cortex in red and green, respectively.

In the final stage, the anatomical labels of the template were propagated to the individual study images using the transformations determined in the final template construction step. Whole and regional brain volume measures were subsequently quantified for each animal. For this purpose, the 39 labels of the NUS atlas were merged into 19 structures of interest: ventricles (i.e. cerebral aquaduct, lateral, third, and fourth ventricles), posterior cerebral cortex, hippocampus, olfactory system, frontal cortex, striatum, thalamus, midbrain, basal ganglia, corpus callosum, amygdala, hypothalamus, cerebellum, pons, medulla, fornix system, anterior commissure, corpora quadrigemina, and internal capsule. An overview of the template construction and labeling steps is provided in Figure 1.

### Data analysis

Data are represented as mean ± standard error of mean (SEM). Analysis was performed with GraphPad Prism 5 software for Windows (GraphPad software Inc., San Diego, California, USA). One-way ANOVA was used in order to compare global group differences (e.g. breeding parameters, total beam crossings in the cage activity, and global and regional brain volumes). Specifically, for SPSN and MWM probe trials (between-subjects variables: group and zone), we used two-way ANOVA. When a time variable was included (e.g. swimming speed in MWM across days), we used two-way repeated measures (RM) ANOVA to analyze the data. Finally, *post hoc* differences were evaluated using the Bonferroni test and the evaluation of percentage of strategy use [relative to chance level (11.11%)] was carried out using a Student's *t*-test. Pearson product mean correlations (r) were calculated to test correlations between behavioral and volumetric data. *P* values obtained by the correlation analysis were corrected for multiple testing using the method of Benjamini and Hochberg (1995). All statistical analyses were performed at a significance level of 0.05.

## Results

### High-dose irradiated mice show decreased cage activity without motor defects

Whole-body irradiation of pregnant mice at E11 did not influence breeding. No significant difference in litter size [*F*_(4, 71)_ = 0.23, *P* = 0.9], male-to-female ratio [*F*_(4, 46)_ = 0.4, *P* = 0.8], and gestation period [*F*_(4, 46)_ = 1.4, *P* = 0.24] could be detected (Table 2).

**Table 2:** **Summary of breeding parameters, body, and brain weight of *in utero* irradiated animals**.

	**Breeding parameters**					
	**0.00 Gy (*N* = 30)**	**0.10 Gy (*N* = 9)**	**0.33 Gy (*N* = 11)**	**0.66 Gy (*N* = 13)**	**1.00 Gy (*N* = 16)**	***F* value**
Litter size	5 ± 0.4	6 ± 0.17	6 ± 0.5	6 ± 0.7	6 ± 0.7	0.4
Male-to-female ratio	1.3 ± 0.26	1.4 ± 0.6	1.7 ± 0.5	1.9 ± 0.6	1.2 ± 0.5	0.4
Gestation period	19 ± 0.11	19 ± 0.14	20 ± 0.20	19 ± 0.16	19 ± 0.13	1.4
	**Weight (g)**					
	**0.00 Gy (*N* = 24)**	**0.10 Gy (*N* = 15)**	**0.33 Gy (*N* = 4)**	**0.66 Gy (*N* = 5)**	**1.00 Gy (*N* = 8)**	***F* value**
Body weight	21 ± 0.22	20 ± 0.3	21 ± 0.7	21 ± 0.3	19 ± 0.25^***^	17
Brain weight	0.53 ± 0.009	0.51 ± 0.009	0.53 ± 0.016	0.49 ± 0.011^*^	0.41 ± 0.009^***^	14

Data are expressed as mean ± SEM. The number of litters/animals used is indicated (N). Significant differences compared to control animals are designated with

* P < 0.05 or

*** P < 0.001. Gy, Gray.

**Figure 2:**
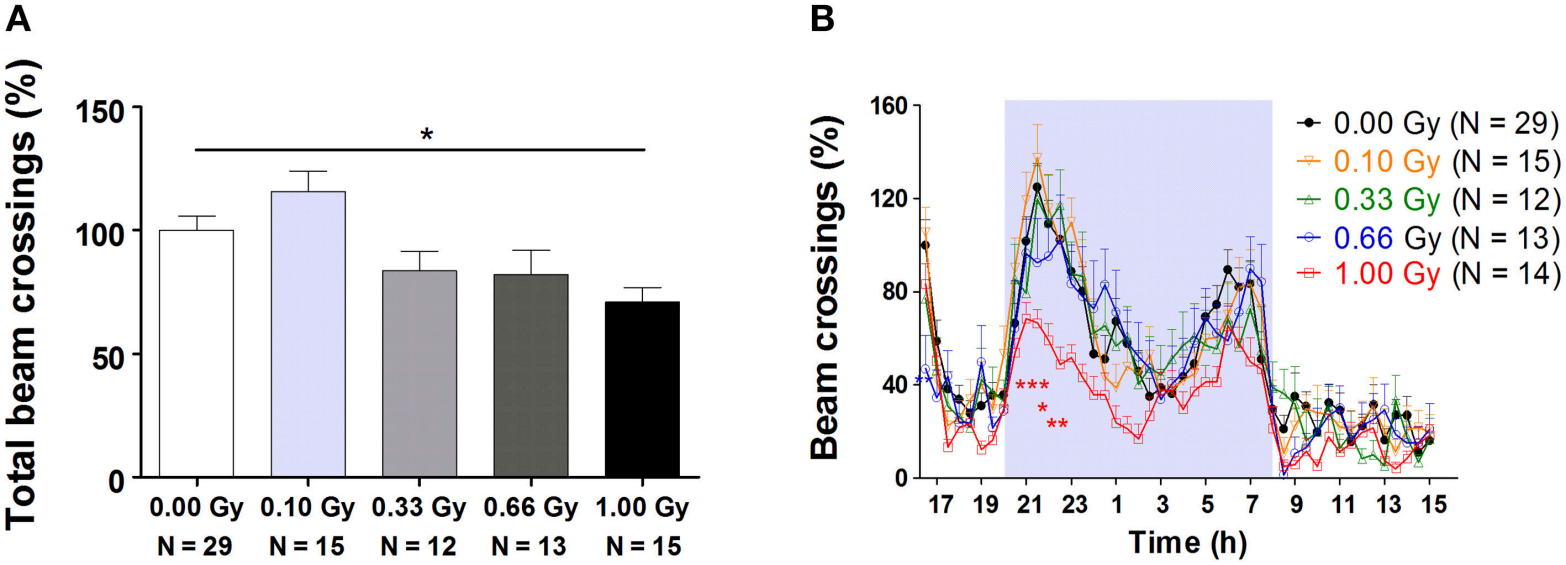
**Decreased 23-h spontaneous cage activity, with the conservation of the circadian rhythm, is observed in prenatally high-dose exposed mice. (A)** Animals irradiated with 1.00 Gy at E11 displayed a decreased activity, as assessed by the total amount of beam crossings during a 23-h period. **(B)** Examination of the circadian profile of all animals indicated a decline in beam crossings during the dark phase in high-dose exposed mice. Data are presented as mean ± SEM. Horizontal lines with an asterisks indicate significant differences between means of two groups. ^*^P < 0.05, ^**^P < 0.01, ^***^P < 0.001. The number of animals used is indicated in the graphs (N). E, embryonic day; Gy, Gray.

Between 4 and 15 weeks of age, mice were subjected to an exhaustive behavioral test battery, including neuromotor, exploratory, social, and learning tests. An overview of these tasks is provided in Table 1. Firstly, total 23-h cage activity recording showed a main effect of irradiation [*F*_(4, 78)_ = 5, *P* = 0.0012], with animals irradiated with the highest dose of 1.00 Gy displaying significantly decreased total beam crossings (Figure 2A). Inspection of the overall 23-h activity patterns clearly showed a main effect of time [*F*_(45, 3510)_ = 34, *P* < 0.0001], meaning that the phasic aspect of the circadian activity cycle was preserved, but also that the amount of beam crossings was significantly affected by irradiation [*F*_(4, 3510)_ = 4, *P* = 0.007] (Figure 2B). During the first peak of the dark phase, a dose-dependent reduction in activity could be seen, especially at the highest dose (Figure 2B). In addition, 0.66-Gy irradiated mice displayed hypoactivity during the first half hour of the test. Thus, prenatal radiation exposure clearly affected spontaneous activity, albeit significantly only in high-dose irradiated mice (≥0.66 Gy).

In the rotarod test, which was used to evaluate mouse motor coordination and equilibrium, we observed a significant effect of trial [*F*_(3, 237)_ = 8, *P* < 0.0001], indicating that animals improved their performance over the four trials, but we did not detect gross radiation-induced alterations in motor functioning [*F*_(4, 237)_ = 1.6, *P* = 0.19]. Also, treadmill analysis of gait parameters could not differentiate between irradiated and non-irradiated animals (Table S1), further corroborating that prenatal radiation exposure did not influence neuromotor abilities at this young adult age.

### Irradiated animals exhibit anxiety-related differences in exploration and sociability

**Figure 3:**
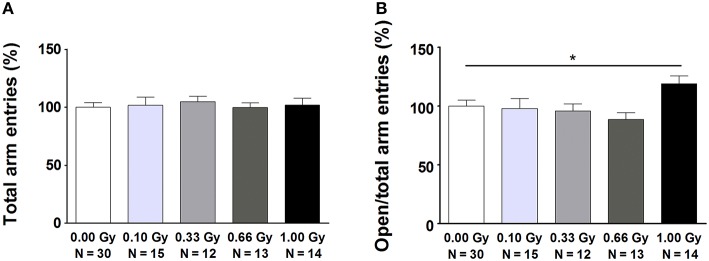
**Mice irradiated *in utero* with 1.00 Gy spent more time in the open arms of the elevated plus maze as compared to sham-exposed animals. (A)** No difference in total arm entries was found. **(B)** When calculating the ratio between the number of entries in the open arms in relation to the total arm entries, a significant increase was seen for the high-dose exposed mice. These results therefore point toward a decreased inhibition of irradiated mice to enter the open arms. Data are presented as mean ± SEM. Horizontal lines with an asterisks indicate significant differences between means of two groups. ^*^P < 0.05. The number of animals used is indicated in the graphs (N). Gy: Gray.

Mouse exploration and social behavior, both tightly associated with anxiety-related responses, were studied using various tests. In the open field and social exploration tasks, no evidence could be found for radiation-induced differences in ambulatory activity (Table S2). In the elevated plus maze, variables that were mainly ambulatory in nature were mostly unchanged as indeed the total number of arm entries [*F*_(4, 79)_ = 0.13, *P* = 1.0] (Figure 3A), open arm entries [*F*_(4, 79)_ = 1.8, *P* = 0.14], closed arm entries [*F*_(4, 79)_ = 0.5, *P* = 0.7], and the percentage of time spent in the open arms [*F*_(4, 79)_ = 0.15, *P* = 1.0] did not differ significantly between groups. However, measures of a more emotional (anxiety-related) nature were altered. In particular, an increased percentage of open arm entries [*F*_(4, 79)_ = 3, *P* = 0.04] (Figure 3B), as well as an increased ratio open/closed arm entries [*F*_(4, 79)_ = 2.8, *P* = 0.03] was observed in animals irradiated with 1.00 Gy at E11 compared to controls and mice irradiated with lower doses. These results suggest anxiety-related defects in exploratory behavior in 1.00-Gy exposed animals.

**Figure 4:**
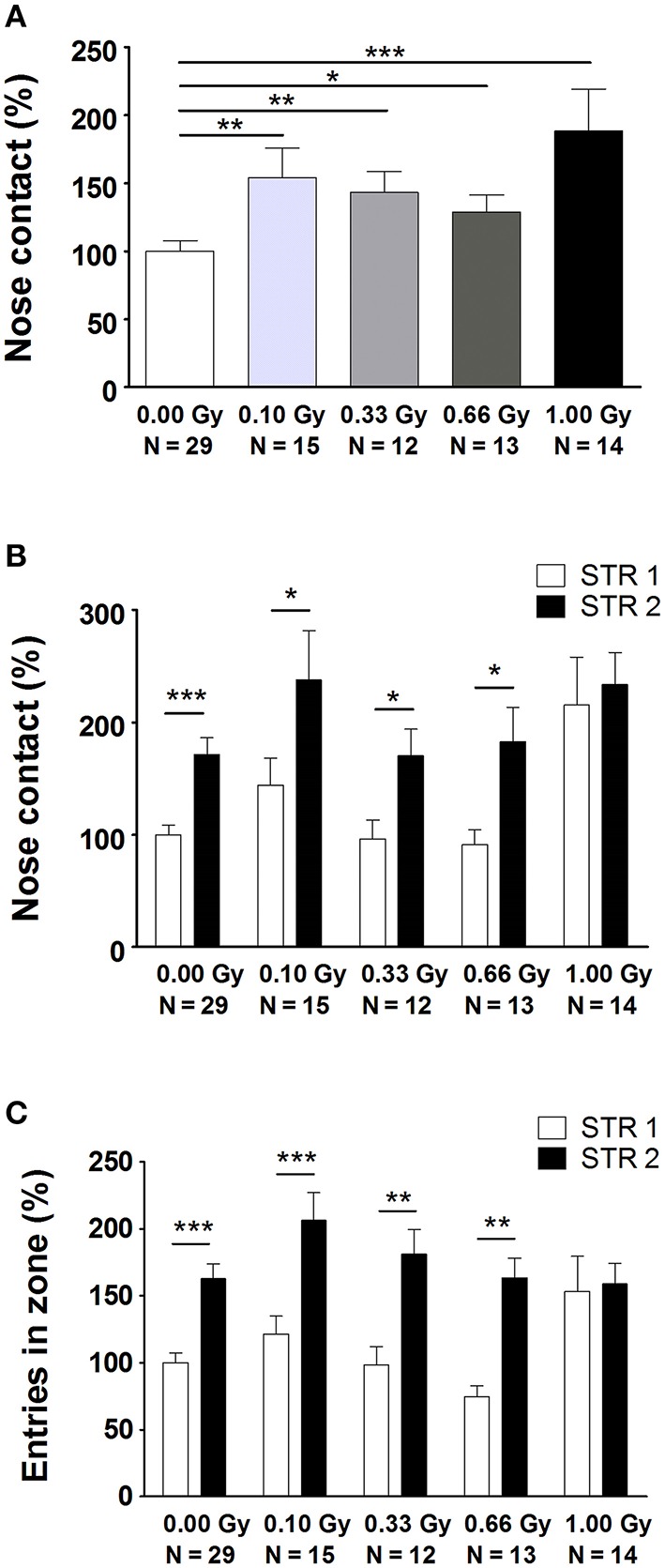
**Prenatally irradiated animals show an increased social behavior, as evidenced by the amount of nose contact in the SPSN task. (A)** During the second trial of the SPSN test, all animals exposed to radiation at E11 engaged more time in nose contact with the STR in the wire cage as compared to sham-irradiated animals. **(B,C)** In the following trial, a second STR, STR2, was placed in the other wire cage and time spent in contact **(B)**, as well as entries in the nose contact zone **(C)**, between STR1 and STR2 were compared. Here, all animals, except for those exposed to 1.00 Gy, showed a significantly increased interest in STR2, indicative for social memory. Data are presented as mean ± SEM. Horizontal lines with an asterisks indicate significant differences between means of two groups. ^*^P < 0.05, ^**^P < 0.01, ^***^P < 0.001. The number of animals used is indicated in the graphs (N). E, embryonic day; Gy, Gray; SPSN, sociability and preference for social novelty; STR, stranger mouse.

Next, several aspects of social performance were analyzed using the SPSN test, which includes measures of sociability and social memory. We unveiled alterations in the time spent in nose contact with STR1 during the sociability trial [*F*_(4, 78)_ = 4, *P* = 0.004] (Figure 4A). Notably, a significant difference was already observed from a dose of 0.10 Gy onwards. In the preference for social novelty trial, we again detected an increase in nose contact (Figure 4B), as well as a difference in the amount of entries in the nose contact zone (Figure 4C). While control animals and animals irradiated with lower doses displayed a clear-cut preference for STR2 as compared to STR1, 1.00-Gy irradiated mice did not. This was shown by a significant effect of STR [*F*_(1, 156)_ = 19, *P* < 0.0001] and an effect of irradiation dose [*F*_(4, 156)_ = 5, *P* = 0.0004] on nose contact (Figure 4B) and on entries in the nose contact zone [effect of STR: *F*_(1, 156)_ = 45, *P* < 0.0001; effect of irradiation dose: *F*_(4, 156)_ = 2.8, *P* = 0.028] (Figure 4C). In summary, SPSN analysis identified profound sociability and social memory changes in mice irradiated with the highest dose at E11. For the latter, 1.00-Gy exposed mice displayed no differentiation between STR1 and STR2, whereas all other groups did.

### Learning and memory is affected in mice prenatally exposed to radiation

**Figure 5:**
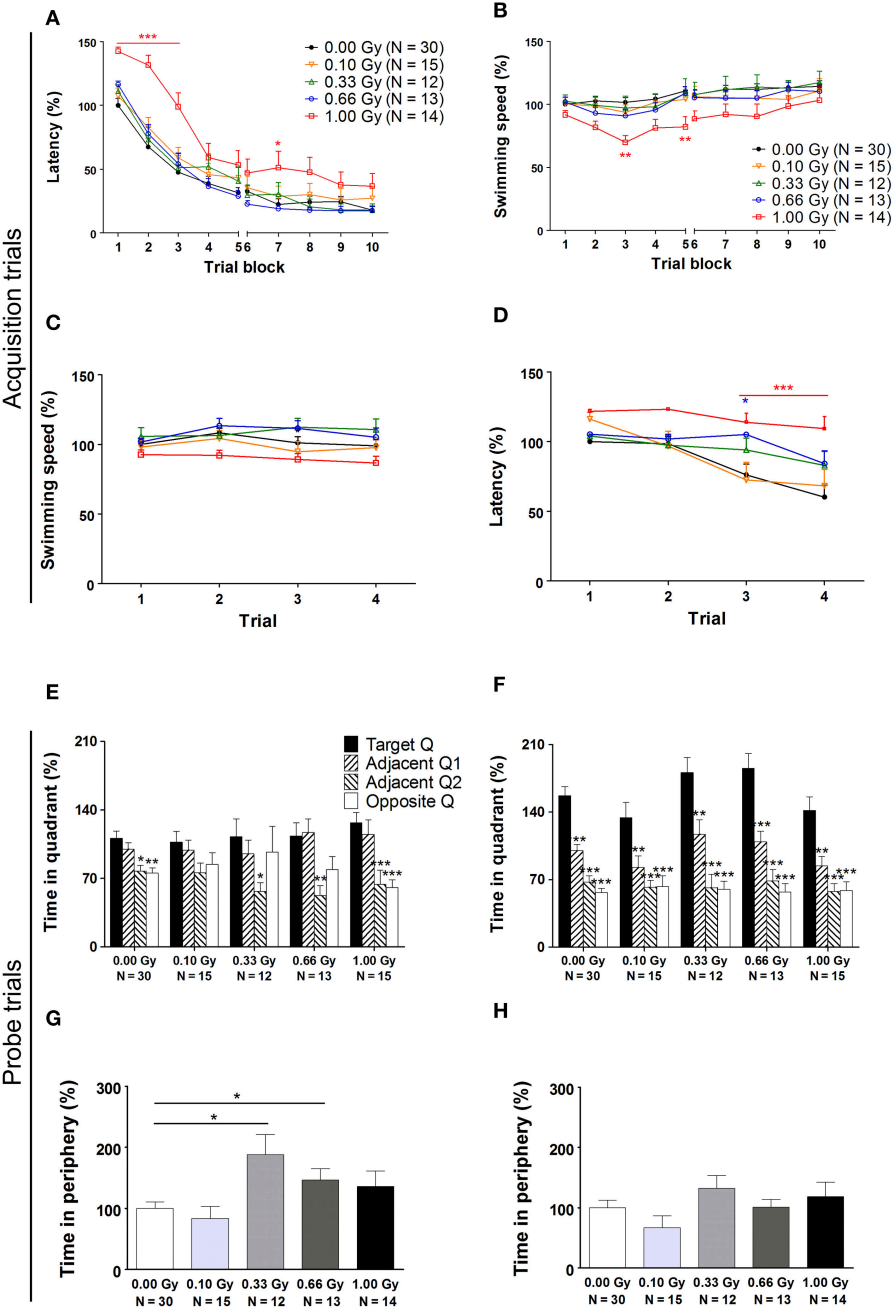
**A traditional hidden-platform MWM analysis points toward aberrations in learning and memory in mice irradiated at E11**. **(A)** A 10-day MWM protocol was exploited to evaluate changes in spatial learning and memory between control and *in utero* irradiated mice. This revealed an increased latency of 1.00-Gy exposed animals to find the hidden platform, which was especially apparent during the first trial days of the test. **(B)** During these trials, the high-dose exposed mice displayed a lower swimming speed. **(C,D)** More careful examination of the first trial day showed that significant changes in escape latency were already visible in 0.66- and 1.00-Gy irradiated animals **(C)**, while the decreased swimming velocity in high-dose irradiated animals was not that evident yet **(D)**. **(E,F)** The probe trials performed on day 6 **(E)** and 11 **(F)** did not indicate a pronounced effect of irradiation on quadrant preference. Asterisks indicate significant differences in time spent in the respective quadrants in respect to the target quadrant for each experimental group. **(G,H)** Notably, mice exposed to a moderate irradiation dose showed an elevated time in swimming in the periphery during the probe trial at day 6 **(G)**, but this difference disappeared in the day 11 probe trial **(H)**. Data are presented as mean ± SEM. ^*^P < 0.05, ^**^P < 0.01, ^***^P < 0.001. The number of animals used is indicated in the graphs (N). E, embryonic day; Gy, Gray; MWM, Morris water maze; Q, quadrant.

Spatial learning and memory of prenatally irradiated mice was assessed using the MWM. In a first instance, we analyzed general parameters such as escape latency and swim speed, but we also included more detailed measures of actual spatio-cognitive performance that are less influenced by neuromotor proficiency (e.g. related to swimming). First, we found a significant effect of training day on latency to find the hidden platform [*F*_(9, 711)_ = 178, *P* < 0.0001], showing that all mice improved their performance as a result of learning (Figure 5A). Notably, 1.00-Gy exposed mice displayed a marginally decreased swimming speed [*F*_(4, 711)_ = 2.2, *P* = 0.08] (Figure 5B), which may have negatively influenced the latency values of these mice. Indeed, irradiation dose affected escape latency [*F*_(4, 711)_ = 5, *P* = 0.0022], as high-dose irradiated animals displayed an increased latency to find the platform, especially during the first three training days. Importantly, altered escape latency was already obvious during the first training day [irradiation dose effect on latency: *F*_(4, 237)_ = 4, *P* < 0.0001] (Figure 5C), in the absence of significant differences in swimming [irradiation dose effect on swim speed: *F*_(4, 237)_ = 3, *P* = 0.020] (Figure 5D). In all, the escape latency learning curve of the high-dose irradiated mice (Figure 5A) showed a clear delay in improving performance over trials and therefore, the reduced swimming velocity of these mice during the subsequent trial days might be attributed to a reduced motivation to swim.

On day 6 and 11 of the experiment, a probe trial was performed to assess the animal's memory for the spatial location of the platform. During the first probe trial, we did not find a dose effect [*F*_(4, 316)_ = 0.009, *P* = 1.0] (Figure 5E). During the second probe trial, target quadrant preference became more pronounced [*F*_(3, 316)_ = 93, *P* < 0.0001] and the dose effect on time spent in the quadrants was significant [*F*_(4, 316)_ = 2.9, *P* = 0.022]. However, in all conditions, mice spent significantly more time in the target quadrant as compared to the other quadrants (Figure 5F), thus suggesting that differences between groups were minor. Furthermore, during the first probe trial, but not during the second, the swimming speed was decreased in mice irradiated with a dose of 0.33 Gy onwards [probe trial 1: *F*_(4, 79)_ = 3, *P* = 0.022; probe trial 2: *F*_(4, 79)_ = 1.5, *P* = 0.22]. Our data thus suggest that moderate-to-high dose exposure significantly affected swimming velocity, as already observed previously for the high-dose irradiated animals during the MWM acquisition trials.

**Figure 6:**
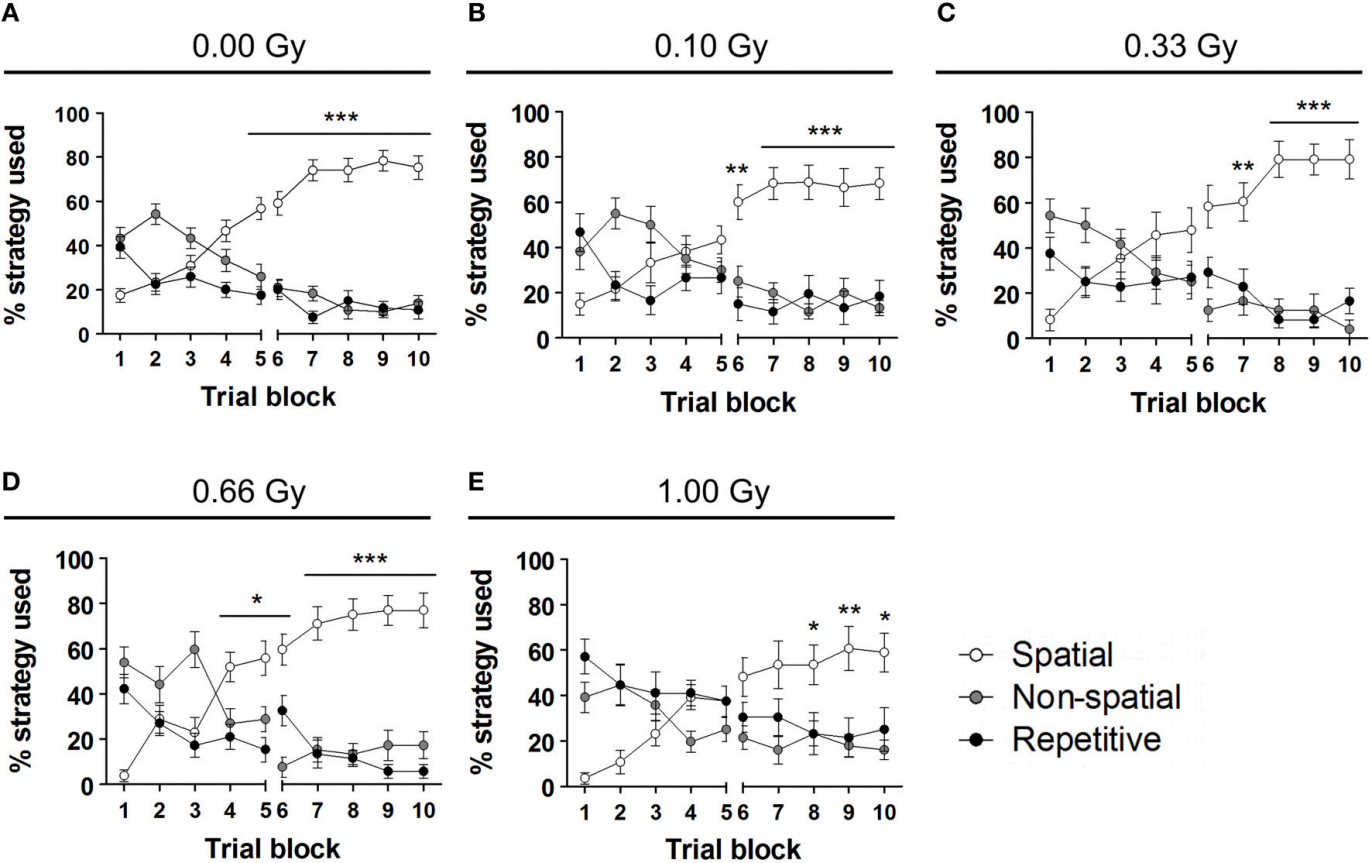
**Swim strategy analysis discloses differences in higher cognitive functioning between dose groups. (A–E)** We used the analysis of swim strategies in the MWM acquisition trials to uncover subtle dissimilarities in higher spatio-cognitive performance in control animals **(A)** vs. mice *in utero* exposed to 0.10 **(B)**, 0.33 **(C)**, 0.66 **(D)**, or 1.00 Gy **(E)**. The exclusive use of the spatial strategy in sham-exposed mice from day 5 onwards was not found in the other dose groups. Indeed, mice irradiated with 0.10 Gy only showed this predominant spatial strategy use from training day 6 on and 0.33-Gy irradiated mice from day 7 on. The most pronounced differences in this analysis were observed in the animals exposed to the highest dose of 1.00 Gy (exclusive use of spatial strategy from day 8 on). Conflicting to this dose-response relationship, the 0.66-Gy exposed animals already significantly used their spatial strategy as compared to the other two strategy classes from training day 4 onwards. Data are presented as mean ± SEM. Horizontal lines with an asterisks indicate significant differences between the spatial vs. non-spatial and repetitive strategies for all the trial blocks underneath. ^*^P < 0.05, ^**^P < 0.01, ^***^P < 0.001. Gy, Gray; MWM, Morris water maze.

To investigate whether or not prenatal irradiation affected actual complex learning abilities and spatio-cognitive performance, we included several analyses that controlled for possible interference of non-cognitive effects on acquisition and probe trial performance. Firstly, we noted that during the first, but not the second, probe trial prenatally irradiated mice spent more time in the periphery of the pool [probe trial 1: *F*_(4, 79)_ = 4, *P* = 0.005; probe trial 2: *F*_(4, 79)_ = 1.6, *P* = 0.18] (Figures 5G,H). Secondly, we assessed the use of spatial cognitive strategies that animals deployed to locate the hidden platform. Taking the three main categories of swim strategies into account (i.e. spatial, non-spatial, and repetitive), we found that control animals increasingly used spatial search strategies (i.e. employing spatial navigational cues), assumed to be the most efficient and cognitively demanding strategies (Lo et al., 2013, 2014). Statistical analysis confirmed this differential use of strategies in the controls [*F*_(2, 783)_ = 70, *P* < 0.0001] (Figure 6A) and further indicated that these spatial strategies were the predominant strategies from training day 5 onwards. In the 0.10-, 0.33-, and 0.66-Gy exposed groups, a similar increased use of spatial strategies was observed [*F*_(2, 378)_ = 16, *F*_(2, 297)_ = 12, and *F*_(2, 324)_ = 59 for the 0.10-, 0.33-, and 0.66-Gy irradiated animals, *P* < 0.0001], but *post hoc* analysis revealed minor changes in the exact timing at which the spatial strategies were utilized in a predominant way (Figures 6B–E). Mice irradiated with 0.10 Gy mostly used the spatial strategies starting from trial day 6 (Figure 6B), 0.33-Gy irradiated animals did this from day 7 (Figure 6C). Unexpectedly, the mice exposed to 0.66 Gy predominantly utilized the spatial strategies already from training day 4 onward (Figure 6D). Inferior learning abilities of the high-dose exposed mice, as observed by altered learning curves, were confirmed using these methods [*F*_(2, 351)_ = 2.1, *P* = 0.14], with the exclusive use of spatial strategies only starting from trial day 8 (Figure 6E). Overall, our analysis thus indicates a dose-dependent delay in the initiation of the predominant use of spatial strategies during the acquisition trials of the MWM, with minor differences already detectable between controls and 0.10-Gy exposed mice.

**Figure 7:**
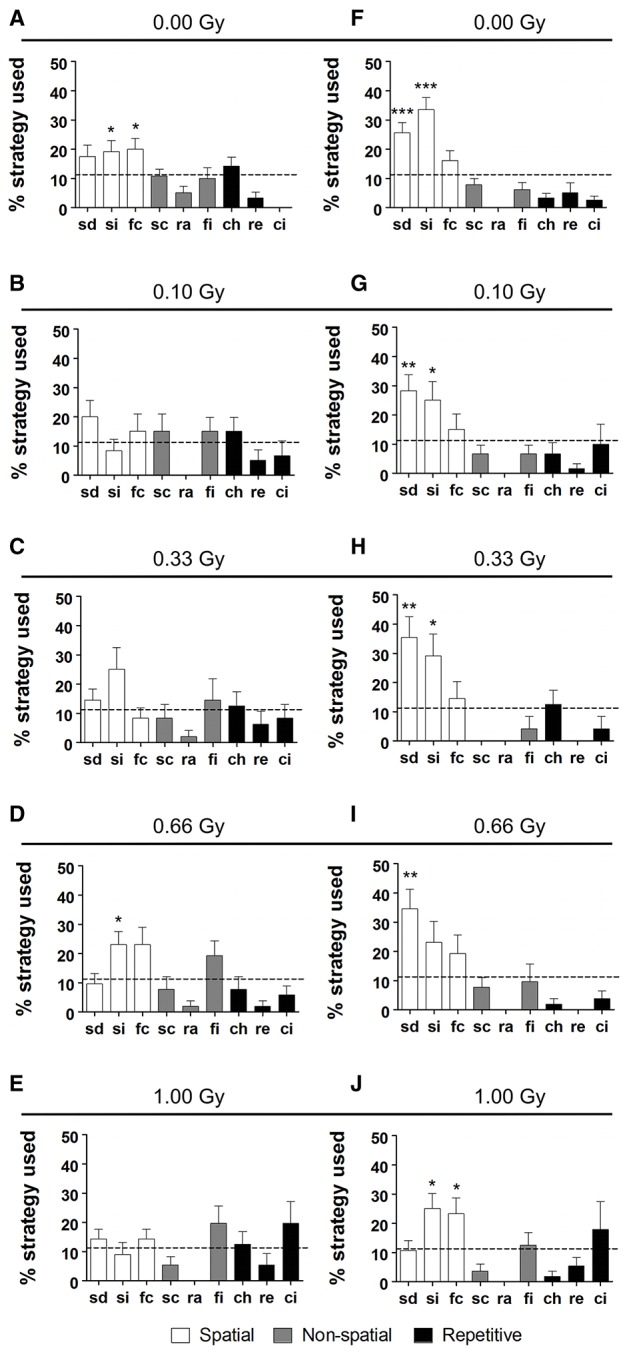
**Irradiated mice have differential search strategy profiles halfway and at the end of MWM training. (A–J)** Low-dose effects on spatial memory were confirmed when detailed strategy profiles were examined at day 5 **(A–E)** and 10 **(F–J)** of the MWM acquisition training. Whereas controls had a significant preference for spatial strategies on training day 5, this was not observed for the other doses, except for the 0.66-Gy irradiated animals that preferentially used the “spatial indirect” strategy. At training day 10, the two highest dose groups (0.66 and 1.00 Gy) still showed alterations in their preference profiles as compared to the sham-exposed condition, with only a significant use of “spatial direct” for the 0.66-Gy group and a significant use of both the “spatial indirect” and “focal correct” strategies in the 1.00-Gy exposed mice. Data are presented as mean ± SEM. ^*^P < 0.05, ^**^P < 0.01, ^***^P < 0.001. ch, chaining; ci, circling; fc, focal correct; fi, focal incorrect; Gy, Gray; MWM, Morris water maze; pe, peripheral looping; ra, random; sc, scanning; sd, spatial direct; si, spatial indirect.

In addition to a global examination of the strategies used, we analyzed all different substrategies used on training days 5 (Figures 7A–E) and 10 (Figures 7F–J). We observed a preference for particular search strategies by the different groups. When comparing the use of all nine possible strategies to chance level (11.11%), control mice showed a significant preference for “spatial indirect” (*P* = 0.04) and “focal correct” (*P* = 0.022) on training day 5 (Figure 7A), whereas this was not the case for the other conditions (Figures 7B–E). None of the irradiated animals had a significant preference for any of the strategies, except for the 0.66-Gy exposed animals that preferred the “spatial indirect” strategy (*P* = 0.020) (Figure 7D). On training day 10, the mice irradiated with 0.10 or 0.33 Gy showed a rather similar strategy preference profile as the control animals, with a significant use of “spatial direct” (*P* = 0.0004, *P* = 0.006, and *P* = 0.006 for the 0.00-, 0.10-, and 0.33-Gy irradiated mice, respectively) and “spatial indirect” (*P* < 0.0001, *P* = 0.05, and *P* = 0.03 for the 0.00-, 0.10-, and 0.33-Gy irradiated mice, respectively) (Figures 7F–H). In the mice exposed to 0.66 Gy, only the “spatial direct” strategy was preferentially used (*P* = 0.004) (Figure 7I). In contrast, the 1.00-Gy irradiated animals preferred the “spatial indirect” (*P* = 0.020) and “focal correct” strategies (*P* = 0.05) (Figure 7J), which is rather similar to the use of strategies in controls at training day 5, which highlights the reduced learning ability in these high-dose exposed mice.

In conclusion, these data uncover differences between controls and irradiated mice in learning abilities and spatio-cognitive performance. High-dose prenatal irradiation induced rather clear deficits in spatial learning and memory, while animals irradiated with a low dose of 0.10 Gy exhibited a moderately inferior spatial memory.

### Behavioral deficits correlate to changes in whole-brain, posterior and frontal cortex, striatum, cerebellum, and ventricular volume assessed by MR imaging

Irradiated animals were smaller in size when compared to controls, quantified as a decreased body weight [*F*_(4, 61)_ = 17, *P* < 0.0001] (Table 2) that persisted throughout life. Notably, 1.00-Gy irradiated mice exhibited a body weight of about 90% in comparison to controls at 50 weeks of age (data not shown). Moreover, brain weight was found to be smaller in both the 0.66- and 1.00-Gy exposed mice [*F*_(4, 28)_ = 20, *P* < 0.0001] (Table 2).

**Figure 8:**
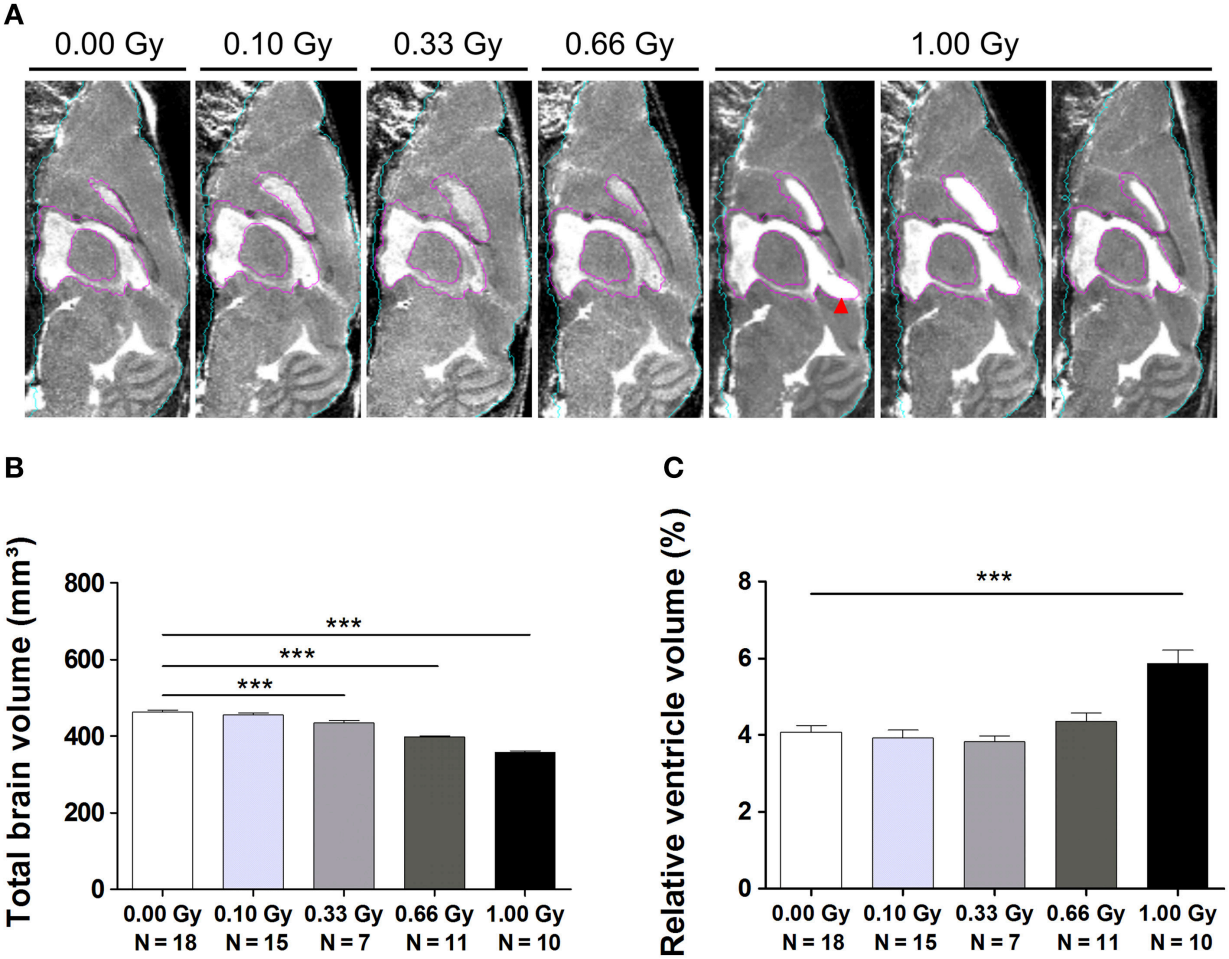
**MRI unveils microcephaly and enlarged ventricles in *in utero* irradiated mice. (A)** MIPs of representative MR images for all dose groups. The quality of atlas-based label propagation was visually verified using these MIPs. Whole-brain outline is shown in cyan, the delineation of the lateral and third ventricles is presented in purple. Animals irradiated with 1.00 Gy typically demonstrated a swelling of the dorsal part of the third ventricle (red arrowhead). **(B)** Total brain volume decreased drastically depending on the radiation dose, with a significant decline already observed in 0.33-Gy exposed mice. **(C)** Ventricles of animals prenatally exposed to 1.00 Gy were increased in size in relation to the total brain volume. Data are presented as mean ± SEM. Horizontal lines with an asterisks indicate significant differences between means of two groups. ^***^P < 0.001. The number of animals used is indicated in the graphs (N). Gy: Gray. MIP: maximum intensity projection. MRI: magnetic resonance imaging.

In order to study this microcephalic phenotype more comprehensively, we performed atlas-guided analysis of brain MR images to quantify the volumes of the whole brain and various subregions in control and irradiated mice (Figure S2). The consistency of this approach was verified by visual inspection across all subjects (Figure 8A). After the exclusion of 11 images with artifacts (Figure S3), absolute and normalized brain volume measures of 19 structures from 61 mouse brain MR images were quantified (Tables 3, 4). We discovered a decline in whole-brain volume in animals exposed to ≥0.33 Gy [*F*_(4, 56)_ = 108, *P* < 0.0001] (Figure 8B), as opposed to a general decrease in brain weight that could only be detected in 0.66- and 1.00-Gy irradiated mice. When we normalized the total brain volume to the body weight, we found a decrease in brain-to-body ratio significant for the 0.66- and 1.00-Gy exposed animals [*F*_(4, 42)_ = 12, *P* < 0.0001].

**Table 3:** **Absolute volumes of brain regions in prenatally irradiated mice as computed by atlas-based segmentation**.

	**Absolute label volumes (mm**^**3**^**)**						
	**0.00 Gy (*N* = 18)**	**0.10 Gy (*N* = 15)**	**0.33 Gy (*N* = 7)**	**0.66 Gy (*N* = 11)**	**1.00 Gy (*N* = 10)**	***F* value**	**%**
Total brain	462.8 ± 4.12	455.5 ± 4.32	434.7 ± 5.38^***^	397.1 ± 2.51^***^	357.2 ± 3.63^***^	108	+23
Ventricles	18.9 ± 0.89	17.9 ± 0.96	16.6 ± 0.75	17.3 ± 0.87	21.0 ± 1.31	1.6	
Posterior cerebral cortex	105.3 ± 1.51	102.1 ± 0.90	97.9 ± 1.09^**^	85.0 ± 1.12^***^	73.8 ± 1.21^***^	98	+30
Hippocampus	23.2 ± 0.25	23.8 ± 0.25	22.3 ± 0.35	20.2 ± 0.29^***^	18.3 ± 0.31^***^	61	+21
Olfactory system	48.2 ± 0.48	46.3 ± 0.50^*^	45.5 ± 1.02^*^	42.9 ± 0.36^***^	39.2 ± 0.56^***^	40	+19
Frontal cortex	16.7 ± 0.28	16.1 ± 0.23	15.6 ± 0.26^*^	13.9 ± 0.20^***^	11.4 ± 0.19^***^	65	+31
Basal ganglia	9.6 ± 0.14	9.4 ± 0.14	9.1 ± 0.11	8.5 ± 0.06^***^	7.8 ± 0.11^***^	31	+19
Striatum	20.9 ± 0.23	20.5 ± 0.18	19.9 ± 0.34	17.3 ± 0.18^***^	14.3 ± 0.22^***^	140	+32
Corpus callosum	10.2 ± 0.18	10.3 ± 0.14	9.8 ± 0.20	8.9 ± 0.11^***^	7.9 ± 0.20^***^	32	+23
Amygdala	14.7 ± 0.20	14.3 ± 0.13	13.5 ± 0.16^**^	12.0 ± 0.21^***^	11.4 ± 0.20^***^	55	+22
Hypothalamus	8.1 ± 0.10	8.1 ± 0.09	7.8 ± 0.08	7.3 ± 0.05^***^	6.9 ± 0.11^***^	30	+15
Thalamus	18.2 ± 0.13	18.0 ± 0.22	17.4 ± 0.21^*^	16.0 ± 0.18^***^	14.0 ± 0.22^***^	81	+23
Midbrain	28.7 ± 0.32	29.1 ± 0.36	27.3 ± 0.36	26.1 ± 0.19^***^	23.5 ± 0.26^***^	46	+18
Cerebellum	50.4 ± 0.70	49.8 ± 0.50	46.0 ± 0.59^***^	39.8 ± 0.36^***^	33.7 ± 0.31^***^	143	+33
Pons	28.8 ± 0.97	29.6 ± 0.34	29.1 ± 0.40	27.4 ± 0.15	25.3 ± 0.27^**^	6	+12
Medulla	24.8 ± 0.45	24.4 ± 0.51	22.8 ± 0.48	21.8 ± 0.36^***^	19.9 ± 0.41^***^	18	+20
Fornix system	5.0 ± 0.12	5.0 ± 0.13	4.8 ± 0.10	4.8 ± 0.06	4.2 ± 0.18^***^	7	+17
Anterior commissure	3.4 ± 0.05	3.2 ± 0.05^*^	3.2 ± 0.06	3.0 ± 0.04^***^	2.6 ± 0.05^***^	37	+24
Corpora quadrigemina	15.7 ± 0.23	15.6 ± 0.18	14.8 ± 0.32	14.0 ± 0.13^***^	12.2 ± 0.25^***^	40	+22
Internal capsule	5.1 ± 0.14	5.3 ± 0.07	4.8 ± 0.13	4.5 ± 0.07^*^	4.2 ± 0.11^***^	14	+18

The column “%” indicates the % increase or decrease in volume between the mice exposed to 1.00 Gy and controls in case of a linear dose-response relationship. Data are expressed as mean ± SEM. The number of animals used is indicated (N). Significant differences compared to control animals are designated with

* P < 0.05,

** P < 0.01, or

*** P < 0.001. Gy: Gray.

**Table 4:** **Normalized average volumes (% of whole-brain volume) of prenatally irradiated mice as computed by atlas-based segmentation**.

	**Normalized label volumes (%)**						
	**0.00 Gy (*N* = 18)**	**0.10 Gy (*N* = 15)**	**0.33 Gy (*N* = 7)**	**0.66 Gy (*N* = 11)**	**1.00 Gy (*N* = 10)**	***F* value**	**%**
Ventricles	4.1 ± 0.18	3.9 ± 0.20	3.8 ± 0.15	4.4 ± 0.22	5.9 ± 0.34^***^	12	+44
Posterior cerebral cortex	22.8 ± 0.23	22.4 ± 0.10	22.5 ± 0.13	21.4 ± 0.16^***^	20.7 ± 0.16^***^	23	−9
Hippocampus	5.0 ± 0.05	5.2 ± 0.04	5.1 ± 0.07	5.1 ± 0.05	5.1 ± 0.07	2.8	
Olfactory system	10.4 ± 0.08	10.2 ± 0.06	10.5 ± 0.16	10.8 ± 0.08^*^	11.0 ± 0.16^***^	11	+5
Frontal cortex	3.6 ± 0.05	3.5 ± 0.04	3.6 ± 0.04	3.5 ± 0.05	3.2 ± 0.04^***^	10	−11
Basal ganglia	2.1 ± 0.024	2.1 ± 0.017	2.1 ± 0.015	2.2 ± 0.019^*^	2.2 ± 0.029^*^	5	+5
Striatum	4.5 ± 0.05	4.5 ± 0.03	4.6 ± 0.05	4.4 ± 0.03	4.0 ± 0.03^***^	23	−12
Corpus callosum	2.2 ± 0.03	2.3 ± 0.021	2.2 ± 0.03	2.2 ± 0.022	2.2 ± 0.04	0.7	
Amygdala	3.2 ± 0.04	3.1 ± 0.03	3.1 ± 0.04	3.0 ± 0.05^*^	3.2 ± 0.04	2.7	
Hypothalamus	1.8 ± 0.016	1.8 ± 0.012	1.8 ± 0.015	1.8 ± 0.016^**^	1.9 ± 0.023^***^	16	+10
Thalamus	3.9 ± 0.024	4.0 ± 0.021	4.0 ± 0.016	4.0 ± 0.027	3.9 ± 0.05	2.2	
Midbrain	6.2 ± 0.05	6.4 ± 0.05	6.3 ± 0.03	6.6 ± 0.05^***^	6.6 ± 0.09^**^	8	+6
Cerebellum	10.9 ± 0.09	10.9 ± 0.04	10.6 ± 0.07	10.0 ± 0.07^***^	9.4 ± 0.04^***^	71	−13
Pons	6.2 ± 0.20	6.5 ± 0.04	6.7 ± 0.05	6.9 ± 0.05^**^	7.1 ± 0.09^***^	7	+14
Medulla	5.4 ± 0.08	5.4 ± 0.09	5.2 ± 0.09	5.5 ± 0.08	5.6 ± 0.11	1.4	
Fornix system	1.1 ± 0.025	1.1 ± 0.026	1.1 ± 0.017	1.2 ± 0.014^*^	1.2 ± 0.05	2.9	
Anterior commissure	0.7 ± 0.009	0.7 ± 0.010	0.7 ± 0.009	0.8 ± 0.008	0.7 ± 0.012	4	
Corpora quadrigemina	3.4 ± 0.04	3.4 ± 0.020	3.4 ± 0.04	3.5 ± 0.04	3.4 ± 0.07	1.5	
Internal capsule	1.1 ± 0.03	1.2 ± 0.013	1.1 ± 0.028	1.1 ± 0.016	1.2 ± 0.025	1.5	

The column “%” indicates the % increase or decrease in volume between the mice exposed to 1.00 Gy and controls in case of a linear dose-response relationship. Data are expressed as mean ± SEM. The number of animals used is indicated (N). Significant differences compared to control animals are designated with

* P < 0.05,

** P < 0.01, or

*** P < 0.001. Gy: Gray.

As expected, the overall reduction in brain volume was associated with a decrease in absolute volume for all of the brain regions (Table 3), except for the ventricles that were comparable in size between experimental groups [*F*_(4, 56)_ = 1.6, *P* = 0.19] (Table 3). The pons showed the smallest decrease in volume, i.e. a 12% decrease in volume between the 1.00-Gy exposed and control animals. The posterior cerebral cortex (30%), frontal cortex (31%), striatum (32%), and cerebellum (33%), on the other hand, showed the largest reduction in volume. Most of the time, differences were only significant after irradiation with a dose of 0.66 and 1.00 Gy, although the posterior cerebral cortex, olfactory system, frontal cortex, amygdala, thalamus, and cerebellum were also significantly reduced in 0.33-Gy irradiated mice (Table 3).

Interestingly, when regional volumes were corrected for variations in individual total brain volume, some additional alterations were revealed. First, irradiation with the highest dose caused a strong increase in normalized ventricle volume [*F*_(4, 56)_ = 11, *P* < 0.0001] (Figure 8C). After examination of the shape of the ventricles in these mice, we noticed that many of the 1.00-Gy exposed animals (±6/10) had an enlarged dorsal protrusion of the third ventricle (Figure 8A), which was not observed in animals from the other dose groups. Next, the normalized volume of the olfactory system [*F*_(4, 56)_ = 11,*P* < 0.0001], basal ganglia [*F*_(4, 56)_ = 5, *P* = 0.0025], hypothalamus [*F*_(4, 56)_ = 16, *P* < 0.0001], midbrain [*F*_(4, 56)_ = 8, *P* < 0.0001], and pons [*F*_(4, 56)_ = 7, *P* = 0.0002] showed a dose-dependent increase (Table 3). Further, the normalized volume of different brain structures was reduced dose-dependently. This was the case for the frontal [*F*_(4, 56)_ = 10, *P* < 0.0001] and posterior cerebral cortex [*F*_(4, 56)_ = 22, *P* < 0.0001], the striatum [*F*_(4, 56)_ = 23, *P* < 0.0001], and the cerebellum [*F*_(4, 56)_ = 71, *P* < 0.0001] (Table 4). Of note, some unexpected significant variations in normalized brain volumes were detected for the amygdala [*F*_(4, 56)_ = 3, *P* = 0.04] and fornix system [*F*_(4, 56)_ = 3, *P* = 0.03]. These alterations were unanticipated since changes in these volumes were not linearly related to the dose. For instance, 0.66-Gy exposed animals exhibited a decrease in relative amygdala volume (*P* = 0.04), while the animals prenatally irradiated with 0.10 (*P* = 0.4), 0.33 (*P* = 0.3), or 1.00 Gy (*P* = 0.6) did not (Table 3).

**Figure 9:**
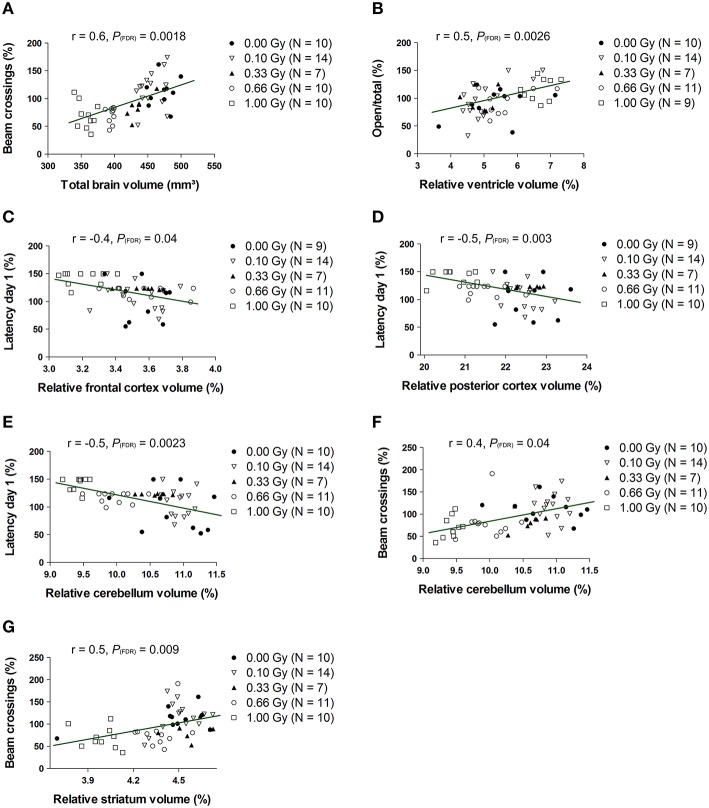
**The whole-brain volume, along with the normalized volumes of the ventricles, frontal and posterior cerebral cortex, cerebellum and striatum, correlate significantly with altered behavioral outcome**. Statistically significant correlations were found between the total brain volume and number of beam crossings in the cage activity **(A)** and between the normalized ventricle volume and the number of open/total **(B)** and open/closed (not shown) arm entries in the elevated plus maze. Furthermore, we discovered correlative relationships between the normalized frontal cortex volume and MWM latency **(C)**, between the normalized posterior cortical volume and MWM latency **(D)**, between the normalized cerebellar volume and MWM latency **(E)**, between the normalized cerebellar volume and cage activity beam crossings **(F)** and between the normalized striatal volume and cage activity beam crossing **(G)**. Correlation coefficients (r) and *P* values are indicated for each correlation. The number of animals used is indicated in the legend (N). FDR, False discovery rate; Gy, Gray; MWM, Morris water maze; SPSN, Sociability and preference for social novelty.

To test whether changes in volume of either the whole brain or specific structures were related to behavioral deficits in these prenatally irradiated animals, we performed correlation analyses. To this end, we used the total brain volume and normalized volumes of brain regions that differed between dose groups in a linear dose-related manner and that constituted more than 3% of the total brain volume. Thus, the absolute whole-brain volume (in mm^3^) and the normalized volumes of the ventricles, posterior and frontal cerebral cortex, striatum, cerebellum, olfactory system, midbrain, and pons (in % of total brain volume) were included in the analysis (Table 5). We found one strong relationship between brain size and behavior, namely between total beam crossings in the cage activity and total brain volume (Figure 9A). Apart from the overall brain volume, various regional volumes were also significantly associated with behavioral parameters. Of these, the normalized volume of the ventricles showed a correlation with open/total (Figure 9B) and open/closed arm entries in the elevated plus maze, whereas the normalized frontal cortex volume showed a significant negative correlation with the escape latency to find the hidden platform during training day 1 of the MWM (Figure 9C). Changes in training day 1 latency also correlated significantly to the decrease in relative posterior cerebral cortex volume (Figure 9D) and the decrease in normalized cerebellar volume (Figure 9E). Further, the cage activity analysis correlated to cerebellum (Figure 9F) and striatum volume (Figure 9G). The normalized volume of the olfactory system, midbrain, and pons did not significantly relate to behavioral outcome. Thus, it appears that the change in both relative regional volumes and whole-brain volume significantly affected behavioral parameters in the cage activity, elevated plus maze, and/or MWM.

**Table 5:** **Correlations between behavioral variables and MRI-based volumetric data**.

	**Volume**					
	**Whole brain (mm^3^)**	**Ventricles (%)**	**Posterior cerebral cortex (%)**	**Frontal cortex (%)**	**Striatum (%)**	**Cerebellum (%)**
Cage activity: beam crossings (%)	0.6^**^	−0.3	0.28	0.19	0.5^**^	0.4^*^
Elevated plus maze: Open/total (%)	−0.09	0.5^**^	−0.3	−0.4	−0.21	−0.21
Elevated plus maze: Open/closed (%)	−0.05	0.5^**^	−0.3	−0.4^*^	−0.22	−0.20
SPSN: Sociability (%)	−0.3	0.06	−0.3	−0.07	−0.07	−0.28
SPSN: Social memory (%)	0.16	−0.3	0.19	0.3	0.3	0.20
MWM: Latency day 1 (%)	−0.29	0.3	−0.5^*^	−0.4^*^	−0.20	−0.5^**^

Behavioral parameters included: (1) the total amount of beam crossings during 23 h in the cage activity, (2) the number of open vs. total arm entries in the elevated plus maze, (3) the number of open vs. closed arm entries in the elevated plus maze, (4) the amount of time spent in nose contact (sociability) during the second trial of the SPSN, (5) the relative amount of time spent in nose contact between STR2 and STR1 (social memory) in the third trial of the SPSN, and (6) the escape latency of animals to find the hidden platform during the first trial day of the MWM acquisition training. Other parameters were evaluated (MWM escape latency on trial day 5 and 10, MWM swimming speed on trial day 1, 5, and 10, MWM % spatial strategy used on trial day 1, 5, and 10), but none of these correlated significantly with the volumetric measures and were therefore not included in the table. Similarly, olfactory system, midbrain, and pons volumes did not correlate significantly with behavioral alterations. Significant correlations are cited with

* P < 0.05 or

** P < 0.01.

MWM, Morris water maze; MRI, magnetic resonance imaging; SPSN, sociability and preference for social novelty; STR, stranger mouse.

In conclusion, cross-sectional changes in volume measurements were found, with prenatal radiation-induced microcephaly apparent in mice irradiated with a relatively low dose of 0.33 Gy. This microcephalic phenotype only related significantly to the decreased cage activity, while variations in normalized volume of the ventricles, frontal and posterior cerebral cortex, striatum, and cerebellum also correlated to various behavioral parameters.

## Discussion

Exposure to high doses of ionizing radiation can be detrimental to human health (Bugher, 1957). The prenatal brain is particularly sensitive to such radiation-related adverse health consequences, with the timing at which irradiation takes place being a decisive factor in the disturbance of development (Schull et al., 1990; Otake and Schull, 1998). Epidemiological research substantiated *in utero* radiation-induced late neurological deficits and provided evidence that these defects can be caused by exposure to relatively low doses, i.e. around 0.10 Gy (Otake et al., 1996). Animal models were developed to relate threshold irradiation to specific outcomes (Kimler and Norton, 1988; Hossain and Uma Devi, 2001; Buratovic et al., 2014) and to try to comprehend high- and low-dose consequences at a cellular and molecular level (Verheyde et al., 2006; Samari et al., 2013; Kempf et al., 2014). Notwithstanding these research efforts on *in vivo* and *in vitro* models, results have been inconsistent and the occurrence of unpredicted effects (Kadhim et al., 2013) hampered the determination of a clear radiation dose threshold, as well as the prediction of low-dose consequences. Despite these uncertainties, the use of radiation in medical diagnosis and treatment, especially the widespread use of CT scans, rises steadily (Smith-Bindman et al., 2008). Although it is widely accepted that caution is warranted in exposing pregnant women to radiation (Ratnapalan et al., 2008), common medical practice in this patient population still includes procedures that exploit low doses of radiation (Osei and Faulkner, 2000; Van Calsteren and Amant, 2014), further emphasizing the need to understand effects of prenatal exposure to low-dose radiation.

Here, we examined the functional and structural sequelae of *in utero* radiation exposure of the mouse at E11 to various doses, ranging between 0.10 and 1.00 Gy. For this purpose, we used behavioral testing and MR imaging. Animals were subjected to a test battery of eight commonly-used behavioral tests to evaluate clinically relevant behavioral components such as emotionality, exploration, and cognition. This allowed us to assess radiation-induced alterations in 23-h spontaneous cage activity, anxiety-related exploration, different elements of social performance, and spatial learning and memory. Whereas, emotional responding and MWM behavior were altered in 1.00-Gy exposed mice, this was also the case in mice irradiated with the lowest dose, 0.10 Gy. The search strategy classification scheme described by Brody and Holtzman (2006) permitted us to inspect the MWM performance in a qualitative manner. We found that all mice showed a clear preference of spatial search strategies at the end of acquisition. Hence, even animals irradiated with a high dose at E11 showed the ability to solve complex tasks and deploy spatio-cognitive strategies, clearly pointing toward a high degree of cognitive preservation despite the irradiation. However, we also discovered that the onset of preferred use of spatial strategies was delayed in an irradiation dose-dependent manner. Such impaired spatio-cognitive performance cannot be reduced to non-cognitive defects, as indicated by the strategy analysis, which is not subjected to mouse performance (e.g. swimming speed). Of particular interest is that these findings appear rather similar to epidemiological data, in which prenatally irradiated humans were found to be compromised in demanding cognitive tasks and scholastic abilities (Schull and Otake, 1999). Such higher cognitive functioning of the brain thus seems to be particularly sensitive to the effects of *in utero* radiation exposure.

Cognitive defects following prenatal irradiation have been observed earlier. Many authors, including ourselves, have reported on radiation-induced learning and memory deficits in various types of mazes (Sienkiewicz et al., 1994, 1999; Devi et al., 1999; Baskar and Devi, 2000; Zhou et al., 2011; Tomášová et al., 2012; Verreet et al., 2015). However, the impaired spatio-cognitive processing in mice exposed to 0.10 Gy is rather unexpected. To our knowledge, only one study, in 1995, has reported on defective maze performance in mice prenatally irradiated with 0.05 Gy (Wang and Zhou, 1995), using internal exposure to β-radiation from tritiated water. Therefore, our study revives the low-dose issue and urges the investigation of doses below 0.10 Gy. Furthermore, we disclosed changes in emotional behavior, of which altered nose contact in the sociability trial of the SPSN in 0.10-Gy exposed mice is suggestive for decreased social inhibition. As emotional actions have never been studied before in prenatally irradiated animals, it is unclear whether this finding could be associated to the previously observed increased aggressive behavior in male mice exposed to 1.00 Gy at E14 (Minamisawa et al., 1992) or possibly to the reduced anxiety-related exploration as observed in our elevated plus maze experiment. There are ample indications for an anxiolytic radiation effect, exemplified by a reduced aversion of *in utero* irradiated animals to a brightly-lit arena (Devi et al., 1999; Baskar and Devi, 2000; Hossain and Uma Devi, 2000; Kiskova and Smajda, 2006). Surprisingly, however, these studies reported on such a reduced anxiety-related exploration in the open field test, in which we found no significant differences. We further unveiled a reduction in cage activity in mice exposed to 1.00 Gy, particularly during the night. Whereas, some studies found increased activity in *in utero* irradiated animals (Kiskova and Smajda, 2006; Tomášová et al., 2012; Verreet et al., 2015), others also reported on decreased activity (Baskar and Devi, 2000; Hossain and Uma Devi, 2000; Zhou et al., 2011). Yet again, this was mainly found in the open field exploration test, in which we did not observe significant changes between conditions. Such variability in results might presumably be explained by inter-laboratory variation in behavioral testing, but differences due to the timing of irradiation in different studies cannot be excluded either. Such inter-laboratory variation also became evident when comparing results with our earlier findings (Verreet et al., 2015). The most striking difference is the former observation of an impaired rotarod performance (Verreet et al., 2015), indicative for neuromotor deficits, which could not be reproduced in this study. One possible explanation for this apparent discrepancy is the use of different genders in both studies. Here, we chose to use female mice since these can be group-housed while group-housing of male animals can provoke aggressive interactions that may lead to negative effects both on the wellbeing of the animals and on the validity of experimental results. Behavioral differences between male and female mice have been reported (Võikar et al., 2001), but have, to our knowledge, never been investigated in *in utero* irradiated animals. Given that gender differences indeed exist in the occurrence and severity of altered behavioral parameters due to cranial irradiation to the P14 mouse (Roughton et al., 2012), a further in-depth examination might be meaningful.

Changes in brain volume are a widely used indicator of pathology and are typically observed in neurodegenerative disorders, including Alzheimer (Grand'maison et al., 2013) and Huntington disease (Zhang et al., 2010). To detect such changes, an MR image atlas-based registration and segmentation approach can be applied (Maheswaran et al., 2009). We used this methodology to interrogate cross-sectional differences between sham-irradiated and irradiated animals *in vivo*. Previously, Saito et al. have repeatedly employed multiple MRI paradigms to assess architectural and circulatory variations in the prenatally irradiated brain, which showed reduced brain size, increased ventricle size, decreased cell density and microvasculature alterations after exposure to high-dose radiation (Saito et al., 2011, 2012, 2014, 2015). Saito and his colleagues were the first to show the excellent applicability of MR imaging in the evaluation of radiation damage to the brain, but global and local volumetry of the prenatally irradiated mouse brain had never been attempted before. While body and brain weight measurements only evidenced growth retardation in the 0.66- and 1.00-Gy exposed mice, we revealed a significant decrease in total brain volume from a dose of 0.33 Gy onwards when applying the volumetric approach. As anticipated, this coincided with a 12–33% decrease in volume between control and 1.00-Gy irradiated mice for all subregions except the ventricles. However, relative to the total brain size, the ventricles in animals exposed to 1.00 Gy were 44% larger, while many brain regions were even reduced in size relative to the smaller irradiated brains.

To determine a relationship between behavior and whole-brain and regional volumes, correlation analyses were performed. A clear relationship between total brain volume and total beam crossings could be established, as well as between the striatum and cerebellum volume and cage activity, which is in accordance with a striatal and cerebellar role in locomotor function (Kravitz et al., 2010; Bazian et al., 2011). The increase in ventricle size related to elevated plus maze outcome. Further, besides an additional link of the posterior cerebral cortex and cerebellum with training day 1 latency, the frontal cortex also correlated to MWM behavior. The prefrontal cortex plays an essential part in rodent MWM learning and memory (Woolley et al., 2013), as hippocampal-prefrontal interconnection is necessary for the animal's navigation in physical space (Pooters et al., 2015). At E11, corticogenesis is initiated (Lodato et al., 2015) and a perturbation of this phenomenon might critically alter cortical volumes. Indeed, both frontal and posterior cerebral cortical volumes were decreased and correlated strongly with MWM escape latency. Moreover, the significant correlation between escape latency and cerebellum volume might be explained by the cerebellar involvement in cognitive aspects unrelated to its role in motor function (Lefort et al., 2015). In all, we revealed the contribution of a decreased normalized volume of the posterior and frontal cerebral cortex, striatum, and cerebellum in a subset of altered parameters of the behavioral test battery. Further investigation is warranted into the mediators of these correlations, whereby it is essential to note that these statistical correlations might not be causal to the observed impaired behavioral outcome. For example, a closer look at inflammation (e.g. astrocyte activation) in these brain structures might be worthwhile since a persistent inflammation in the prenatally irradiated brain has been proposed before (Saito et al., 2012; Verreet et al., 2015).

In summary, in the present report, we studied brain functionality and architecture in prenatally irradiated mice. Behavioral tests that included elements of neuromotor performance, exploratory behavior, and learning and memory ability revealed dose-dependent changes in activity, social behavior, anxiety-related exploration, and spatio-cognitive performance. Whereas, alterations in behavior were mostly observed when irradiated with moderate to high doses of radiation, our data also suggest that both emotionality and higher cognitive abilities were significantly altered in mice exposed to 0.10 Gy at E11. We revealed microcephaly from a dose of 0.33 Gy onwards and dedicated MR imaging and volumetry further showed alterations in regional brain volumes of irradiated animals. The absolute volume of the total brain and normalized volumes of the ventricles, frontal cortex, posterior cerebral cortex, striatum, and cerebellum correlated to behavioral parameters. In all, we have thoroughly studied the dose-response relationship of mouse brain function and structure following prenatal irradiation, which unveiled effects at doses previously assumed to be harmless.

## Author contributions

MN and LL were responsible for mouse breeding. TV, JR, AL, and KG analyzed behavioral and MRI data. TV performed all experimental work and drafted the manuscript. JR, RQ, MV, SB, FM, UH, RD, LM, and MB were responsible for the design and critical reviewing the work. All authors read and approved the final manuscript.

### Conflict of interest statement

The authors declare that the research was conducted in the absence of any commercial or financial relationships that could be construed as a potential conflict of interest.

## Abbreviations

ch: Chaining
ci: Circling
E: Embryonic day
fc: Focal correct
FDR: False discovery rate
fi: Focal incorrect
Gy: Gray
MIP: Maximum intensity projection
MRI: Magnetic resonance imaging
MWM: Morris water maze
P: Postnatal day
pe: Peripheral looping
Q: Quadrant
ra: Random
RARE: Rapid acquisition with refocused echoes
sc: Scanning
sd: Spatial direct
si: Spatial indirect
SPSN: Sociability and preference for social novelty
STR: Stranger mouse.

## References

[B1] Aldridge K. Wang L. Harms M. P. Moffitt A. J. Cole K. K. Csernansky J. G.. (2012). A longitudinal analysis of regional brain volumes in macaques exposed to X-irradiation in early gestation. PLoS ONE 7:e43109. 10.1371/journal.pone.0043109 22905212 PMC3419216

[B2] Atwood T. Payne V. S. Zhao W. Brown W. R. Wheeler K. T. Zhu J. M.. (2007a). Quantitative magnetic resonance spectroscopy reveals a potential relationship between radiation-induced changes in rat brain metabolites and cognitive impairment. Radiat. Res. 168, 574–581. 10.1667/RR0735.1 17973545

[B3] Atwood T. Robbins M. E. Zhu J. M. (2007b). Quantitative *in vivo* proton MR spectroscopic evaluation of the irradiated rat brain. J. Magn. Reson. Imaging 26, 1590–1595. 10.1002/jmri.21095 17968883

[B4] Bai J. Trinh T. L. Chuang K. H. Qiu A. (2012). Atlas-based automatic mouse brain image segmentation revisited: model complexity vs. image registration. Magn. Reson. Imaging 30, 789–798. 10.1016/j.mri.2012.02.010 22464452

[B5] Baskar R. Devi P. U. (2000). Influence of gestational age to low-level gamma irradiation on postnatal behavior in mice. Neurotoxicol. Teratol. 22, 593–602. 10.1016/S0892-0362(00)00076-3 10974598

[B6] Bazian A. S. Grigir'ian G. A. Ioffe M. E. (2011). Regulation of motor behaviour. Usp. Fiziol. Nauk. 42, 65–80. 21950009

[B7] Benjamini Y. Hochberg Y. (1995). Controlling the false discovery rate: a practical and powerful approach to multiple testing. J. R. Stat. Soc. B 27, 289–300.

[B8] Brody D. L. Holtzman D. M. (2006). Morris water maze search strategy analysis in PDAPP mice before and after experimental traumatic brain injury. Exp. Neurol. 197, 330–340. 10.1016/j.expneurol.2005.10.020 16309676 PMC1913184

[B9] Bugher J. C. (1957). Radiation and human health. Am. J. Public Health Nations Health 47, 682–687. 10.2105/AJPH.47.6.682 13424812 PMC1551032

[B10] Buratovic S. Stenerlöw B. Fredriksson A. Sundell-Bergman S. Viberg H. Eriksson P. (2014). Neonatal exposure to a moderate dose of ionizing radiation causes behavioural defects and altered levels of tau protein in mice. Neurotoxicology 45, 48–55. 10.1016/j.neuro.2014.09.002 25265567

[B11] Callaerts-Vegh Z. Leo S. Vermaercke B. Meert T. D'Hooge R. (2012). LPA5 receptor plays a role in pain sensitivity, emotional exploration and reversal learning. Genes Brain Behav. 11, 1009–1019. 10.1111/j.1601-183X.2012.00840.x 23039190

[B12] Chan K. C. Khong P. L. Cheung M. M. Wang S. Cai K. X. Wu E. X. (2009). MRI of late microstructural and metabolic alterations in radiation-induced brain injuries. J. Magn. Reson. Imaging 29, 1013–1020. 10.1002/jmri.21736 19388094

[B13] Cleary J. O. Modat M. Norris F. C. Price A. N. Jayakody S. A. Martinez-Barbera J. P.. (2011). Magnetic resonance virtual histology for embryos: 3D atlases for automated high-throughput phenotyping. Neuroimage 54, 769–778. 10.1016/j.neuroimage.2010.07.039 20656039

[B14] Devi P. U. Hossain M. Bisht K. S. (1999). Effect of late fetal irradiation on adult behavior of mouse: Dose-response relationship. Neurotoxicol. Teratol. 21, 193–198. 10.1016/S0892-0362(98)00039-7 10192280

[B15] Doss M. Little M. P. Orton C. G. (2014). Point/Counterpoint: low-dose radiation is beneficial, not harmful. Med. Phys. 41, 070601. 10.1118/1.4881095 24989368 PMC4109571

[B16] File S. E. Seth P. (2003). A review of 25 years of the social interaction test. Eur. J. Pharmacol. 463, 35–53. 10.1016/S0014-2999(03)01273-1 12600701

[B17] Gazdzinski L. M. Cormier K. Lu F. G. Lerch J. P. Wong C. S. Nieman B. J. (2012). Radiation-induced alterations in mouse brain development characterized by magnetic resonance imaging. Int. J. Radiat. Oncol. Biol. Phys. 84, e631–e638. 10.1016/j.ijrobp.2012.06.053 22975609

[B18] Grand'maison M. Zehntner S. P. Ho M. K. Hébert F. Wood A. Carbonell F.. (2013). Early cortical thickness changes predict beta-amyloid deposition in a mouse model of Alzheimer's disease. Neurobiol. Dis. 54, 59–67. 10.1016/j.nbd.2013.02.005 23454197

[B19] Hossain M. Uma Devi P. (2000). Effect of irradiation at the early fetal stage on adult brain function in the mouse: locomotor activity. Int. J. Radiat. Biol. 76, 1397–1402. 10.1080/09553000050151673 11057748

[B20] Hossain M. Uma Devi P. (2001). Effect of irradiation at the early foetal stage on adult brain function of mouse: learning and memory. Int. J. Radiat. Biol. 77, 581–585. 10.1080/09553000110037394 11382336

[B21] Kadhim M. Salomaa S. Wright E. Hildebrandt G. Belyakov O. V. Prise K. M.. (2013). Non-targeted effects of ionising radiation-Implications for low dose risk. Mutat. Res. 752, 84–98. 10.1016/j.mrrev.2012.12.001 23262375 PMC4091999

[B22] Kempf S. J. Casciati A. Buratovic S. Janik D. von Toerne C. Ueffing M.. (2014). The cognitive defects of neonatally irradiated mice are accompanied by changed synaptic plasticity, adult neurogenesis and neuroinflammation. Mol. Neurodegener. 9:57. 10.1186/1750-1326-9-57 25515237 PMC4280038

[B23] Kimler B. F. Norton S. (1988). Behavioral changes and structural defects in rats irradiated *in utero*. Int. J. Radiat. Oncol. Biol. Phys. 15, 1171–1177. 10.1016/0360-3016(88)90200-3 3182350

[B24] Kiskova J. Smajda B. (2006). Behavioural changes in prenatal rats irradiated with low dosage of gamma-rays. Bull. Vet. Inst. Pulawy 50, 595–598.

[B25] Kokošová N. Tomášová L. Kisková T. Šmajda B. (2015). Neuronal analysis and behaviour in prenatally gamma-irradiated rats. Cell. Mol. Neurobiol. 35, 45–55. 10.1007/s10571-014-0144-8 25537960 PMC11486342

[B26] Kovacevic N. Henderson J. T. Chan E. Lifshitz N. Bishop J. Evans A. C.. (2005). A three-dimensional MRI atlas of the mouse brain with estimates of the average and variability. Cereb. Cortex 15, 639–645. 10.1093/cercor/bhh165 15342433

[B27] Kravitz A. V. Freeze B. S. Parker P. R. Kay K. Thwin M. T. Deisseroth K.. (2010). Regulation of parkinsonian motor behaviours by optogenetic control of basal ganglia circuitry. Nature 466, 622–626. 10.1038/nature09159 20613723 PMC3552484

[B28] Lefort J. M. Rochefort C. Rondi-Reig L. Group of L.R.R. is member of Bio-Psy Labex ENP Foundation (2015). Cerebellar contribution to spatial navigation: new insights into potential mechanisms. Cerebellum 14, 59–62. 10.1007/s12311-015-0653-0 25630873

[B29] Likar B. Viergever M. A. Pernus F. (2001). Retrospective correction of MR intensity inhomogeneity by information minimization. IEEE Trans. Med. Imaging 20, 1398–1410. 10.1109/42.974934 11811839

[B30] Lo A. C. De Maeyer J. H. Vermaercke B. Callaerts-Vegh Z. Schuurkes J. A. D'Hooge R. (2014). SSP-002392, a new 5-HT4 receptor agonist, dose-dependently reverses scopolamine-induced learning and memory impairments in C57Bl/6 mice. Neuropharmacology 85, 178–189. 10.1016/j.neuropharm.2014.05.013 24863046

[B31] Lo A. C. Tesseur I. Scopes D. I. Nerou E. Callaerts-Vegh Z. Vermaercke B.. (2013). Dose-dependent improvements in learning and memory deficits in APPPS1-21 transgenic mice treated with the orally active Abeta toxicity inhibitor SEN1500. Neuropharmacology 75, 458–466. 10.1016/j.neuropharm.2013.08.030 24035915

[B32] Lodato S. Shetty A. S. Arlotta P. (2015). Cerebral cortex assembly: generating and reprogramming projection neuron diversity. Trends Neurosci. 38, 117–125. 10.1016/j.tins.2014.11.003 25529141 PMC4334136

[B33] Ma D. Cardoso M. J. Modat M. Powell N. Wells J. Holmes H.. (2014). Automatic structural parcellation of mouse brain MRI using multi-atlas label fusion. PLoS ONE 9:e86576. 10.1371/journal.pone.0086576 24475148 PMC3903537

[B34] MacKenzie-Graham A. Lee E. F. Dinov I. D. Bota M. Shattuck D. W. Ruffins S.. (2004). A multimodal, multidimensional atlas of the C57BL/6J mouse brain. J. Anat. 204, 93–102. 10.1111/j.1469-7580.2004.00264.x 15032916 PMC1571243

[B35] Maes F. Collignon A. Vandermeulen D. Marchal G. Suetens P. (1997). Multimodality image registration by maximization of mutual information. IEEE Trans. Med. Imaging 16, 187–198. 10.1109/42.563664 9101328

[B36] Maheswaran S. Barjat H. Bate S. T. Aljabar P. Hill D. L. Tilling L.. (2009). Analysis of serial magnetic resonance images of mouse brains using image registration. Neuroimage 44, 692–700. 10.1016/j.neuroimage.2008.10.016 19015039

[B37] Minamisawa T. Hirokaga K. Sasaki S. Noda Y. (1992). Effects of fetal exposure to gamma rays on aggressive behavior in adult male mice. J. Radiat. Res. 33, 243–249. 10.1269/jrr.33.243 1464856

[B38] Modat M. Ridgway G. R. Taylor Z. A. Lehmann M. Barnes J. Hawkes D. J.. (2010). Fast free-form deformation using graphics processing units. Comput. Methods Programs Biomed. 98, 278–284. 10.1016/j.cmpb.2009.09.002 19818524

[B39] Mothersill C. Seymour C. (2013). Implications for human and environmental health of low doses of ionising radiation. J. Environ. Radioact. 133, 5–9. 10.1016/j.jenvrad.2013.04.002 23664231

[B40] Naert A. Callaerts-Vegh Z. D'Hooge R. (2011). Nocturnal hyperactivity, increased social novelty preference and delayed extinction of fear responses in post-weaning socially isolated mice. Brain Res. Bull. 85, 354–362. 10.1016/j.brainresbull.2011.03.027 21501666

[B41] Osei E. K. Faulkner K. (2000). Radiation risks from exposure to diagnostic X-rays during pregnancy. Radiography 6, 131–144. 10.1053/radi.2000.0238

[B42] Otake M. Schull W. J. (1998). Radiation-related brain damage and growth retardation among the prenatally exposed atomic bomb survivors. Int. J. Radiat. Biol. 74, 159–171. 10.1080/095530098141555 9712546

[B43] Otake M. Schull W. J. Lee S. (1996). Threshold for radiation-related severe mental retardation in prenatally exposed A-bomb survivors: a re-analysis. Int. J. Radiat. Biol. 70, 755–763. 10.1080/095530096144644 8980673

[B44] Pooters T. Van der Jeugd A. Callaerts-Vegh Z. D'Hooge R. (2015). Telencephalic neurocircuitry and synaptic plasticity in rodent spatial learning and memory. Brain Res. 1621, 294–308. 10.1016/j.brainres.2015.01.015 25619550

[B45] Ratnapalan S. Bentur Y. Koren G. (2008). Doctor, will that x-ray harm my unborn child? CMAJ 179, 1293–1296. 10.1503/cmaj.080247 19047611 PMC2585137

[B46] Roughton K. Kalm M. Blomgren K. (2012). Sex-dependent differences in behavior and hippocampal neurogenesis after irradiation to the young mouse brain. Eur. J. Neurosci. 36, 2763–2772. 10.1111/j.1460-9568.2012.08197.x 22758785

[B47] Rueckert D. Sonoda L. I. Hayes C. Hill D. L. Leach M. O. Hawkes D. J. (1999). Nonrigid registration using free-form deformations: application to breast MR images. IEEE Trans. Med. Imaging 18, 712–721. 10.1109/42.796284 10534053

[B48] Saito S. Aoki I. Sawada K. Suhara T. (2012). Quantitative assessment of central nervous system disorder induced by prenatal X-ray exposure using diffusion and manganese-enhanced MRI. NMR Biomed. 25, 75–83. 10.1002/nbm.1715 21538637

[B49] Saito S. Aoki I. Sawada K. Sun X. Z. Chuang K. H. Kershaw J.. (2011). Quantitative and noninvasive assessment of prenatal X-ray-induced CNS abnormalities using magnetic resonance imaging. Radiat. Res. 175, 1–9. 10.1667/RR2134.1 21175341

[B50] Saito S. Sawada K. Hirose M. Mori Y. Yoshioka Y. Murase K. (2014). Diffusion tensor imaging of brain abnormalities induced by prenatal exposure to radiation in rodents. PLoS ONE 9:e107368. 10.1371/journal.pone.0107368 25202992 PMC4159342

[B51] Saito S. Sawada K. Mori Y. Yoshioka Y. Murase K. (2015). Brain and arterial abnormalities following prenatal X-ray irradiation in mice assessed by magnetic resonance imaging and angiography. Congenit. Anom. (Kyoto) 55, 103–106. 10.1111/cga.12101 25534523

[B52] Samari N. De Saint-Georges L. Pani G. Baatout S. Leyns L. Benotmane M. A. (2013). Non-conventional apoptotic response to ionising radiation mediated by N-methyl D-aspartate receptors in immature neuronal cells. Int. J. Mol. Med. 31, 516–524. 10.3892/ijmm.2013.1245 23338045 PMC3597540

[B53] Sawiak S. J. Wood N. I. Williams G. B. Morton A. J. Carpenter T. A. (2013). Voxel-based morphometry with templates and validation in a mouse model of Huntington's disease. Magn. Reson. Imaging 31, 1522–1531. 10.1016/j.mri.2013.06.001 23835187 PMC3919157

[B54] Schull W. J. Otake M. (1999). Cognitive function and prenatal exposure to ionizing radiation. Teratology 59, 222–226. 10.1002/(SICI)1096-9926(199904)59:4<222::AID-TERA6>3.0.CO;2-M 10331523

[B55] Schull W. J. Norton S. Jensh R. P. (1990). Ionizing radiation and the developing brain. Neurotoxicol. Teratol. 12, 249–260. 10.1016/0892-0362(90)90096-U 2196423

[B56] Selemon L. D. Ceritoglu C. Ratnanather J. T. Wang L. Harms M. P. Aldridge K.. (2013). Distinct abnormalities of the primate prefrontal cortex caused by ionizing radiation in early or midgestation. J. Comp. Neurol. 521, 1040–1053. 10.1002/cne.23217 22911497 PMC3560314

[B57] Sienkiewicz Z. J. Haylock R. G. Saunders R. D. (1994). Prenatal irradiation and spatial memory in mice: investigation of dose-response relationship. Int. J. Radiat. Biol. 65, 611–618. 10.1080/09553009414550701 7910199

[B58] Sienkiewicz Z. J. Haylock R. G. Saunders R. D. (1999). Differential learning impairments produced by prenatal exposure to ionizing radiation in mice. Int. J. Radiat. Biol. 75, 121–127. 10.1080/095530099140889 9972799

[B59] Smith-Bindman R. Miglioretti D. L. Larson E. B. (2008). Rising use of diagnostic medical imaging in a large integrated health system. Health Aff. (Millwood) 27, 1491–1502. 10.1377/hlthaff.27.6.1491 18997204 PMC2765780

[B60] Tomášová L. Smajda B. Sevc J. (2012). Effects of prenatal irradiation on behaviour and hippocampal neurogenesis in adult rats. Acta Physiol. Hung. 99, 126–132. 10.1556/APhysiol.99.2012.2.5 22849836

[B61] Van Calsteren K. Amant F. (2014). Cancer during pregnancy. Acta Obstet. Gynecol. Scand. 93, 443–446. 10.1111/aogs.12380 24628416

[B62] Verheyde J. de Saint-Georges L. Leyns L. Benotmane M. A. (2006). The role of Trp53 in the transcriptional response to ionizing radiation in the developing brain. DNA Res. 13, 65–75. 10.1093/dnares/dsi028 16766514

[B63] Verreet T. Quintens R. Van Dam D. Verslegers M. Tanori M. Casciati A.. (2015). A multidisciplinary approach unravels early and persistent effects of X-ray exposure at the onset of prenatal neurogenesis. J. Neurodev. Disord. 7:3. 10.1186/1866-1955-7-3 26029273 PMC4448911

[B64] Võikar V. Kõks S. Vasar E. Rauvala H. (2001). Strain and gender differences in the behavior of mouse lines commonly used in transgenic studies. Physiol. Behav. 72, 271–281. 10.1016/S0031-9384(00)00405-4 11240006

[B65] Wang B. Zhou X. Y. (1995). Effects of prenatal exposure to low-dose beta radiation from tritiated water on the neurobehavior of mice. J. Radiat. Res. 36, 103–111. 10.1269/jrr.36.103 7473343

[B66] Williams P. M. Fletcher S. (2010). Health effects of prenatal radiation exposure. Am. Fam. Physician 82, 488–493. 20822083

[B67] Woolley D. G. Laeremans A. Gantois I. Mantini D. Vermaercke B. Op de Beeck H. P.. (2013). Homologous involvement of striatum and prefrontal cortex in rodent and human water maze learning. Proc. Natl. Acad. Sci. U.S.A. 110, 3131–3136. 10.1073/pnas.1217832110 23382228 PMC3581978

[B68] Zhang J. Peng Q. Li Q. Jahanshad N. Hou Z. Jiang M.. (2010). Longitudinal characterization of brain atrophy of a Huntington's disease mouse model by automated morphological analyses of magnetic resonance images. Neuroimage 49, 2340–2351. 10.1016/j.neuroimage.2009.10.027 19850133 PMC2929697

[B69] Zhou F. W. Rani A. Martinez-Diaz H. Foster T. C. Roper S. N. (2011). Altered behavior in experimental cortical dysplasia. Epilepsia 52, 2293–2303. 10.1111/j.1528-1167.2011.03267.x 21933180 PMC4364520

