# Supplementary material for: Persistent Impact of In utero Irradiation on Mouse Brain Structure and Function Characterized by MR Imaging and Behavioral Analysis
Source: Front Behav Neurosci. 2016 May 4;10:83. doi: 10.3389/fnbeh.2016.00083 (PMC4854899; doi:10.3389/fnbeh.2016.00083)
Supplement: Supplementary file 1 [file Presentation1.PDF]

frontiers

Supplementary Material

Persistent Impact of *In Utero* Irradiation on Mouse Brain Structure and Function Characterized by MR Imaging and Behavioral Analysis

Tine Verreet, Janaki Raman Rangarajan, Roel Quintens, Mieke Verslegers, Adrian C. Lo, Kristof Govaerts, Mieke Neefs, Liselotte Leysen, Sarah Baatout, Frederik Maes, Uwe Himmelreich, Rudi D'Hooge, Lieve Moons, Mohammed A. Benotmane*

*** Correspondence:** Corresponding Author: rafi.benotmane@sckcen.be

Supplementary Figures and Tables

Supplementary Figures

Main strategy	Subcategory	Description
- Spatial 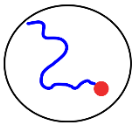	- Spatial direct (sd)	Mice swim to the platform in a straight line
	- Spatial indirect (si)	Mice swim to the plaform with minor deviations from a straight line
	- Focal correct (fc)	Mice search in the target quadrant
- Non-spatial 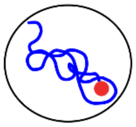	- Scanning (sc)	Mice search in the center of the pool without a quadrant bias
	- Random (ra)	Mice search in the entire pool without a quadrant bias
	- Focal incorrect (fi)	Mice search in a non-target quadrant
- Repetitive 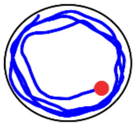	- Chaining (ch)	Mice swim in circles, equidistant from the wall
	- Peripheral looping (pe)	Mice search close to the wall
	- Circling (ci)	Mice swim in many typical circular loops

Figure S1. Overview of the different search strategies that mice can adopt in order to locate the hidden platform in the Morris water maze (MWM).

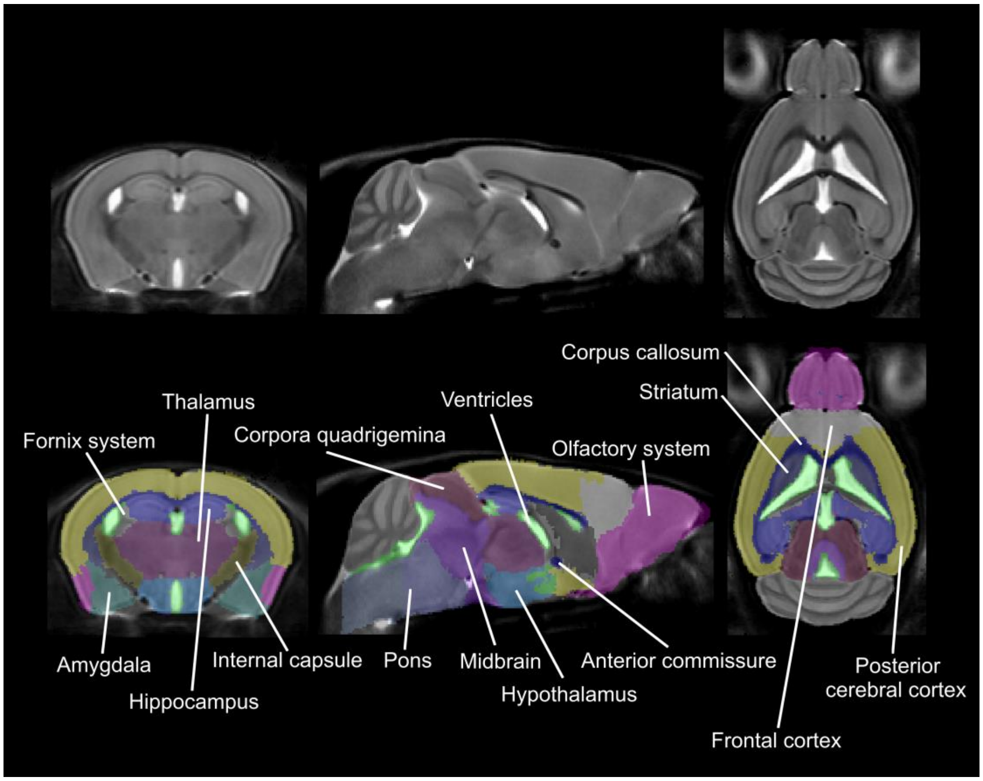

Figure S2. Study-specific mean template. This template was constructed iteratively by non-rigid registration of all available study images (i.e. from all conditions). Anatomical labels were propagated from the NUS template through registration. In our statistical analysis of label volumes, the number of labels was reduced from 39 to 19.

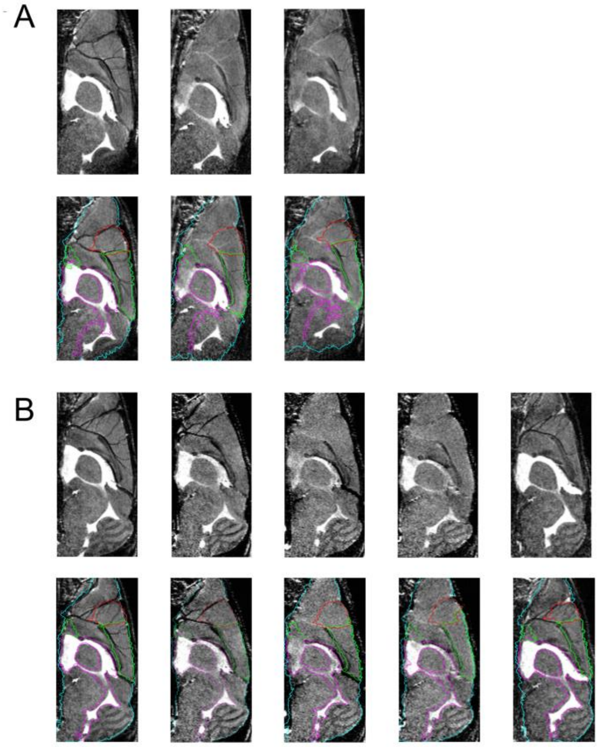

Figure S3. Representative brain MR images used for visual inspection. The accuracy of the atlas-based segmentation was verified by overlaying the propagated atlas labels on the native MRI images. **(A)** Representative images with artefacts that showed mismatch between the labels and underlying anatomy (e.g. along the fourth ventricle, shown in purple). These images were excluded from the statistical analysis. The artifacts presumably resulted from poor signal-to-noise ratio in the caudal part of the brain (cerebellum region). **(B)** Images with anatomical labels that consistently matched the underlying anatomy were considered correct and included in the subsequent analysis. MRI: Magnetic resonance imaging.

Supplementary Tables

Table S1. Overview of gait parameters in irradiated and sham-irradiated animals. The number of animals used is indicated (N). Data are expressed as mean ± SEM. Gy: Gray

	0.00 Gy (N = 27)	0.10 Gy (N = 15)	0.33 Gy (N = 11)	0.66 Gy (N = 12)	1.00 Gy (N = 13)	F value
**Stride length**						
Forelimbs	100 ± 1.1	101 ± 1.1	99 ± 1.6	90 ± 4	94 ± 8	1.7
Hindlimbs	100 ± 1.2	101 ± 1.1	101 ± 1.6	94 ± 2.0	93 ± 7	1.4
**Paw angle**						
Forelimbs	100 ± 73	103 ± 45	97 ± 162	170 ± 53	72 ± 64	0.9
Hindlimbs	100 ± 5	104 ± 6	86 ± 6	91 ± 7	82 ± 8	2.0
**Absolute paw angle**						
Forelimbs	100 ± 9	88 ± 14	128 ± 27	75 ± 15	95 ± 15	1.3
Hindlimbs	100 ± 5	103 ± 5	86 ± 6	91 ± 7	81 ± 8	2.3
**Stance/swing ratio**						
Forelimbs	100 ± 3	103 ± 3	97 ± 4	103 ± 5	111 ± 4	1.7
Hindlimbs	100 ± 1.9	98 ± 2.1	96 ± 4	101 ± 3	95 ± 3	0.9
**Hind limb shared stance time**	100 ± 4	97 ± 4	86 ± 8	91 ± 5	82 ± 7	2.0
**Stance**						
Forelimbs	100 ± 0.9	102 ± 1.5	98 ± 1.9	93 ± 4	98 ± 7	1.1
Hindlimbs	100 ± 1.2	100 ± 1.5	99 ± 1.7	95 ± 2.0	91 ± 7	1.7
**Stride**						
Forelimbs	100 ± 1.1	101 ± 1.1	99 ± 1.6	90 ± 4	94 ± 8	1.6
Hindlimbs	100 ± 1.2	101 ± 1.1	101 ± 1.6	94 ± 2.0	93 ± 7	1.4
**Swing**						
Forelimbs	100 ± 2.4	98 ± 1.7	101 ± 3	86 ± 5	90 ± 9	2.1
Hindlimbs	100 ± 1.9	102 ± 1.3	103 ± 4	97 ± 3	97 ± 8	1.0

Table S2. Ambulatory measures in the open field and social exploration test. The number of animals used is indicated (N). Data are expressed as mean ± SEM. Gy: Gray.

	0.00 Gy (N = 30)	0.10 Gy (N = 15)	0.33 Gy (N = 12)	0.66 Gy (N = 13)	1.00 Gy (N = 14)	F value
**Open field**						
Path length	100 ± 6	108 ± 5	108 ± 9	110 ± 11	106 ± 6	0.4
Mean speed	100 ± 6	108 ± 5	108 ± 9	111 ± 11	106 ± 6	0.4
Line crossings	100 ± 5	103 ± 5	104 ± 8	108 ± 8	99 ± 6	0.3
Center entries	100 ± 10	97 ± 7	107 ± 15	107 ± 14	103 ± 13	0.12
% Path length in center	100 ± 7	94 ± 6	94 ± 7	95 ± 10	96 ± 10	0.13
Latency to first center entry	100 ± 16	126 ± 35	61 ± 23	46 ± 11	61 ± 17	2.2
Time in center	100 ± 11	96 ± 9	101 ± 11	89 ± 16	122 ± 35	0.4
**Social exploration**						
Path length	100 ± 4	114 ± 6	101 ± 8	106 ± 7	108 ± 4	1.2
Mean speed	100 ± 4	114 ± 7	101 ± 8	106 ± 7	108 ± 4	1.2
Line crossings	100 ± 6	118 ± 7	101 ± 7	107 ± 7	118 ± 5	2.0
Center entries	100 ± 8	122 ± 14	103 ± 8	127 ± 9	135 ± 11	2.5
% Path length in center	100 ± 4	104 ± 10	92 ± 5	101 ± 6	109 ± 9	0.7
Latency to first center entry	100 ± 35	171 ± 106	189 ± 70	104 ± 48	62 ± 29	0.7
Time in center	100 ± 4	91 ± 8	92 ± 6	105 ± 5	106 ± 9	1.0
